# Strain-dependent dynamic re-alignment of collagen fibers in skeletal muscle extracellular matrix

**DOI:** 10.1016/j.actbio.2024.08.035

**Published:** 2024-08-30

**Authors:** Ross P. Wohlgemuth, Sathvik Sriram, Kyle E. Henricson, Daryl T. Dinh, Sarah E. Brashear, Lucas R. Smith

**Affiliations:** a Department of Neurobiology, Physiology, & Behavior, University of California Davis, United States; b Department of Physical Medicine and Rehabilitation, University of California Davis, United States

**Keywords:** Skeletal muscle, Extracellular matrix, Collagen architecture, Second harmonic generation, Muscle mechanics

## Abstract

Collagen fiber architecture within the skeletal muscle extracellular matrix (ECM) is significant to passive muscle mechanics. While it is thought that collagen fibers re-orient themselves in response to changes in muscle length, this has not been dynamically visualized and quantified within a muscle. The goal of this study was to measure changes in collagen alignment across a range of muscle lengths and compare the corresponding alignment to muscle mechanics. We hypothesized that collagen fibers dynamically increase alignment in response to muscle stretching, and this change in alignment is related to passive muscle stiffness. Further, we hypothesized that digesting collagen fibers with collagenase would reduce the re-alignment response to muscle stretching. Using DBA/2J and D2.*mdx* mice, we isolated extensor digitorum longus (EDL), soleus, and diaphragm muscles for collagenase or sham treatment and decellularization to isolate intact or collagenase-digested decellularized muscles (DCMs). These DCMs were mechanically tested and imaged using second harmonic generation microscopy to measure collagen alignment across a range of strains. We found that collagen alignment increased in a strain-dependent fashion, but collagenase did not significantly affect the strain-dependent change in alignment. We also saw that the collagen fibers in the diaphragm epimysium (surface ECM) and perimysium (deep ECM) started at different angles, but still re-oriented in the same direction in response to stretching. These robust changes in collagen alignment were weakly related to passive DCM stiffness. Overall, we demonstrated that the architecture of muscle ECM is dynamic in response to strain and is related to passive muscle mechanics.

## Statement of significance

Our study presents a unique visualization and quantification of strain-induced changes in muscle collagen fiber alignment as they relate to passive mechanics. Using dynamic imaging of collagen in skeletal muscle we demonstrate that as skeletal muscle is stretched, collagen fibers re-orient themselves along the axis of stretch and increase their alignment. The degree of alignment and the increase in alignment are each weakly related to passive muscle stiffness. Collagenase treatments further demonstrate that the basis for muscle Extracellular matrix stiffness is dependent on factors beyond collagen crosslinking and alignment. Together the study contributes to the knowledge of the structure-function relationships of muscle extracellular matrix to tissue stiffness relevant to conditions of fibrosis and aberrant stiffness.

## 1. Introduction

Skeletal muscle is a dynamic tissue that undergoes changes in length which affect its passive and active mechanical properties [1,2]. While the structural basis for the active length-tension relationship is well understood, the structural basis for the passive length-tension relationship is largely empirical. It is evident that both intracellular and extracellular components of the muscle determine the passive tension as muscle is strained [3–6], although the mechanical contribution of each component varies. Typically, in mammalian muscle the intracellular contribution to passive mechanics ranges from 25 to 60 %, while the extracellular proportion ranges from 40 to 75 % [7–11]. At the protein level, the primary passive load bearer of the myofibers is titin [4,7,12], while in the extracellular matrix (ECM) it is collagen [5,13]. Although titin and collagen exhibit non-linear mechanical properties, higher orders of muscle tissue i.e. whole muscle and fascicles vs. bundles and single myofibers, exhibit increasing non-linear passive mechanical properties [5,6,14]. This suggests that components of the ECM, which are more abundant in higher orders of muscle tissue, are important to the non-linear passive mechanical properties of skeletal muscle. Since passive muscle mechanical properties affect the range of motion over which muscles can produce active force, elucidating the structural basis of the passive length-tension relationship would be beneficial to understanding the mechanisms behind diseases that are characterized by reduced muscle range of motion, such as cerebral palsy or muscular dystrophies [15,16].

Collagen fibrils exhibit a typical pattern of mechanical behavior [17–23], such that when collagen fibrils are strained they experience deformation on the meso– and microscales. As collagen fibrils are strained from slack length they alter their coarse alignment and undergo stretching to remove their crimped pattern [17,20,21]. Further strain leads to sliding and stretching of the collagen molecules themselves before intermolecular bonds between collagen molecules are broken from supramaximal stresses [17,22,23]. While in normal muscle function collagen fibers are not undergoing mechanical failure, there is evidence that changes in collagen fiber alignment and straightness occur within the collagen matrix as muscle changes length [24–29]. Similarly, the collagen fibers within tendon, which are mechanically connected to the muscle ECM [30], reduce their crimp upon loading and become more aligned [31–34]. Because increases in collagen alignment and straightness are related to increases in collagen fibril stress, the architecture of the muscle collagen matrix is anticipated to influence muscle viscoelastic properties.

Interestingly, there is variation in the alignment of collagen fibers between healthy and fibrotic muscles, and a higher degree of alignment is correlated to increased passive muscle stiffness [35–37]. Taken altogether, it would seem likely that as muscle lengthens the collagen matrix deforms in part by changes in collagen fiber alignment and straightness. Thus, we hypothesized that collagen fibers dynamically re-align in skeletal muscle in response to increased strain. Additionally, we hypothesized that the increase in collagen alignment is related to the increase in ECM-based passive stiffness at higher strains. We tested this hypothesis by collecting hindlimb and diaphragm muscles from DBA/2J (wildtype) and D2.*mdx* mice, a model of Duchenne muscular dystrophy exhibiting more severe fibrosis than other genetic backgrounds [10,35,36,38]. We performed a collagenase or sham treatment on matched left and right muscles (or strips of diaphragm muscle) to see if collagenase treatment would blunt any dynamic changes in collagen architecture that occurred in response to tissue strain. Our method of imaging unfixed decellularized muscles across length scales was implemented to measure dynamic collagen architecture across strains. Our study provides a unique visualization and quantification of strain-dependent changes in collagen architecture that occur in skeletal muscle.

## 2. Materials & methods

### 2.1. Ethical approval

All experiments involving animals were approved by the University of California, Davis Institutional Animal Care and Use Committee under IACUC protocol #22579.

### 2.2. Animal handling and dissection

DBA/2J (wildtype) and D2.B10-Dmd*mdx*/J (D2.*mdx*) mice were cared for in the UC Davis Teaching and Research Animal Care Services facility. Mice were housed on a 12:12 light-dark cycle and provided ad libitum access to food and water. Mice were between 47 and 51 weeks of age at sacrifice (wildtype male *N* = 3, wildtype female *N* = 4; D2.*mdx* male *N* = 6, D2.*mdx* female *N* = 4). We refer to this age group of mice as middle-aged, although the D2.*mdx* mouse and other models of DMD have shortened lifespans [39–42]. Mice were anesthetized with 2.5 % Isoflurane gas in 1 L/min oxygen. Soleus and extensor digitorum longus (EDL) were collected from the hindlimbs while the mice were under anesthesia. After hindlimb muscles were obtained, cervical dislocation was done while mice were still under anesthesia. Following cervical dislocation, the liver and diaphragm were collected. Soleus, EDL, and diaphragm muscles were placed in a bath of oxygenated Ringer’s solution (Sodium Chloride, Potassium Chloride, Calcium Chloride dihydrate, Potassium Phosphate Monobasic, Magnesium Sulfate, 4-(2-Hydroxyethyl)piperazine-1-ethanesulfonic acid (HEPES), Glucose) while the liver was flash frozen in liquid nitrogen and stored at −70 °C.

### 2.3. Measurement of optimum length

Soleus, EDL, and diaphragm muscles were prepared for passive mechanical testing as previously described [10,35,36]. Briefly, 7–0 sutures were cinched at the muscle-tendon junctions of the soleus and EDL muscles. Six strips of diaphragm muscle cut along the myofiber angle were tied with 7–0 suture at the central tendon and ribs. Suture loops were attached to the 300C-LR-Dual-Mode motor arm and force transducer (Aurora Scientific) in a bath of 28 °C oxygenated Ringer’s solution. The optimum length for isometric force contraction (Lo) was calculated using a series of twitches as the muscle was incrementally stretched using the 701C stimulator (Aurora Scientific). The muscle length at which the most force was produced from contraction was set as the Lo length. This length was measured with calipers between the sutures at either muscle-tendon junction. Physiological cross-sectional area (PCSA) was calculated using the muscle length (Lm), mass (m), ratio of fiber length to Lo (Lf/Lo) and standard density of muscle (*ρ* = 1.06 g/cm^3^; PCSA = *m*/Lo^∗^(Lf/Lo)^∗^*ρ*) [43]. Following measurement of Lo, soleus, EDL, and two strips of diaphragm muscle were sequestered for collagenase/sham and decellularization treatments while the remaining four strips of diaphragm muscle underwent passive mechanical testing before and after a collagenase/sham treatment.

### 2.4. Collagenase and decellularization protocols

Muscle collagenase digestion and decellularization were performed and validated in previous reports [10,11]. Both left and right EDL and soleus muscles received a collagenase or sham treatment and a subsequent decellularization treatment. Of the six diaphragm strips isolated, two strips received either a collagenase or sham treatment with a subsequent decellularization treatment. Of the remaining four diaphragm strips, two strips were pinned at slack length and two were pinned at 112.5 % of Lo (12.5 % strain). Each pair of strips underwent either a collagenase or sham treatment prior to subsequent mechanical testing. Collagenase or sham digestion was performed on EDL, soleus, and diaphragm muscles. To make the stock collagenase solution, type II collagenase (Gibco, Carlsbad, CA) was added to 1 ml of Hank’s Balanced Salt Solution with calcium, magnesium, and no phenol red (Gibco, Carlsbad, CA) and mixed. Type II collagenase cleaves the bond between a neutral amino acid (X) and glycine in the sequence Pro-X-Gly-Pro, which occurs at a high frequency within collagen molecules, specifically within the backbone. Cleaving these bonds structurally compromises collagen molecules, reducing their mechanical properties. Collagenase stock solution was then diluted, stored, and applied to muscles as previously described [10]. Following collagenase or sham treatment, muscles were then decellularized in line with previous descriptions [10,11].

### 2.5. Passive mechanical testing

Passive mechanical testing was carried out in a similar manner to previous studies [10,35,36,44–46]. Decellularized EDL, soleus, and diaphragm muscles were placed in a bath of Ringer’s solution prior to passive mechanical testing. Suture loops on decellularized muscles (DCMs) were placed on the 300C-LR-Dual-Mode motor arm and force transducer (Aurora Scientific) in a bath of 28 °C Ringer’s solution and were set to a length of 85 % Lo (−15 % strain). DCMs underwent a passive mechanical protocol that included preconditioning and held stretch steps. The preconditioning steps occurred before each held stretch step and consisted of lengthening the DCM by 5 % Lo at a rate of 1 Hz for 5 s. The held stretch steps consisted of lengthening the DCM by 5 % Lo at a rate of 1 Lo/s beyond the current length and held at this length for 120 s. Preconditioning and held stretch steps were continued up to 95, 100, 105, 110, and 115 % of Lo (−10 %, −5 %, 0 %, 5 %, 10 %, and 15 % strain). The stiffness (Young’s modulus) was calculated as the slope of the quadratic fit of nominal stress (passive tensile force per PCSA) and strain values. Dynamic stiffness was the slope of the fit with the maximum nominal stress values over the held stretch steps while elastic stiffness was the slope of the fit with the nominal stress values measured at the end of the 120 second held stretch step. Elastic index was calculated as the ratio between elastic and dynamic stiffness at each strain. DCM samples that mechanically failed prior to 10 % strain were not used for comparisons of passive mechanics. DCM samples that exhibited an elastic index greater than 1 were removed from comparisons involving elastic index. DCM samples were placed in storage solution (125 mM K-propionate, 20 mM Imidazole, 5 mM Ethylene Glycol-bis(*β*-aminoethyl ether)-N,N,N’,N’-tetraacetic acid (EGTA), 2 mM Magnesium Chloride Hexahydrate, 2 mM Adenosine-5-triphosphate Disodium Salt in 50 % glycerol; pH=7.00) [47] until used for second harmonic generation imaging.

Intact strips of diaphragm muscle underwent similar mechanical testing protocols before and after collagenase or sham digestion. However, the starting length for these protocols was 100 % Lo, and the preconditioning and held stretch steps for intact muscles covered 2.5 % increments from 102.5 % to 105, 107.5, 110, and 112.5 % of Lo (2.5 %, 5 %, 7.5 %, 10 %, 12.5 % strain). Following collagenase or sham treatment, diaphragm muscles were mechanically tested from 100 % Lo (0 % strain) in 2.5 % increments up to 120 % of Lo (20 % strain). Stiffness and elastic index were measured similar to the DCM samples.

### 2.6. Second harmonic generation imaging for collagen architecture

**Fig. 1.**
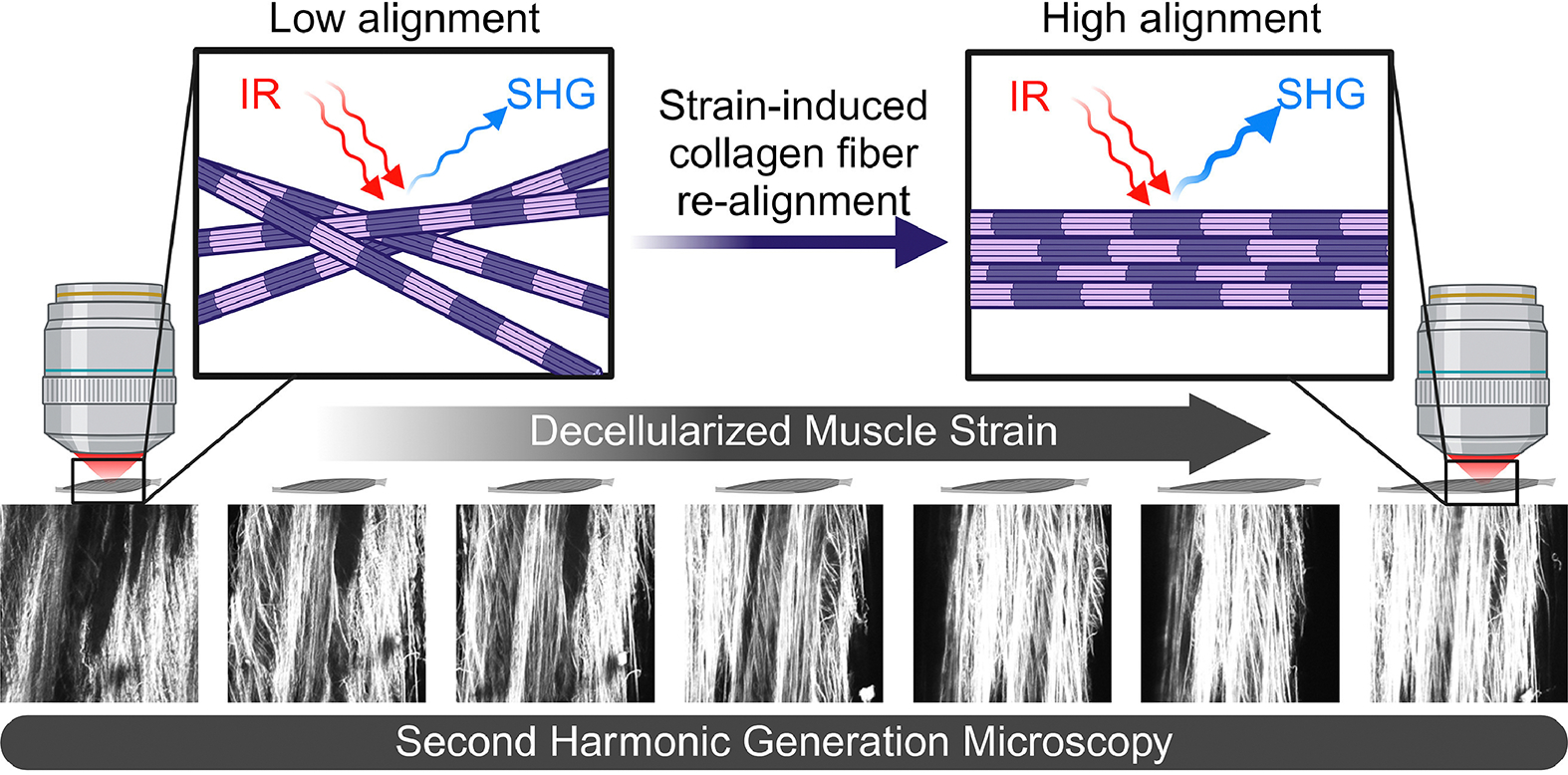
Schematic of dynamic second harmonic generation (SHG) imaging technique. SHG microscopy was performed on decellularized muscles across a range of strains that matched the mechanical testing protocol. Collagen fibers within the decellularized muscles upconverted two incident infrared (IR) photons to one photon of their second harmonic (half the wavelength). Collagen fibers visibly increased their alignment as the muscle was strained. Highly aligned collagen fibers exhibit more intense SHG signal [49,50]. Figure created with BioRender.com.

Second harmonic generation (SHG) microscopy was performed at the Advanced Imaging Facility in the UC Davis School of Veterinary Medicine using a Leica TCS SP8 fit with a Mai Tai deep see laser. A 25× water immersion objective was used with a multiphoton laser. Briefly, decellularized muscles (DCMs) were removed from storage solution and placed in a bath of phosphate buffered saline (PBS) at room temperature. Using the previously tied sutures on each DCM, looped sutures were placed around hooks in an apparatus similar to that used in the mechanical testing protocols. DCMs were set to −15 % strain in line with the mechanical testing protocol. Each sample was imaged in a single location along the muscle belly using the multiphoton laser tuned to 848 nm for an image stack size of at least 50 μm with a slice thickness of 1 μm. During image analysis, we noticed that the architecture of the diaphragm epimysium was noticeably different from the perimysium, prompting us to analyze the layers separately. Since the majority of image stacks included the top surface of the muscle, we were able to compare the epimysium and perimysium in most diaphragm DCMs. However, we found that none of our images for the WT collagenase-treated diaphragm DCMs included signal from the epimysial layer, so only the perimysial architecture for this group was measured. Following collection of the first image stack, DCMs were passively strained in 5 % increments from −15 % to 15 % and imaged in the same location for each sample length (Fig. 1). Following imaging, DCMs were washed in PBS and then flash frozen in liquid nitrogen. DCMs that mechanically failed or had sutures slip off the muscle belly were either retied and/or analyzed only until the corresponding length at failure. Custom MATLAB scripts and processing in ImageJ were used to analyze collagen fiber diameter, area fraction, and alignment in the images stacks as previously described [35,36,48]. Intrinsic alignment index was calculated as the alignment of collagen fibers relative to the mean collagen fiber angle. Axial alignment index was calculated as the alignment of collagen fibers relative to the axis of strain (or angle of strain), which was set to 90° for all SHG images. Axial deviation angle was calculated as the absolute value of the difference between the mean collagen angle and angle of strain. Collagen fiber diameter and area fraction data for each muscle were averaged over the image stacks across the range of strains.

### 2.7. Hydroxyproline and collagen solubility assay

Total collagen content and cross-linking were measured in EDL, soleus, and diaphragm DCMs as well as the liver using the hydroxyproline and collagen solubility assay as described in other studies [10,35,36,46,51]. Briefly, flash frozen samples were powdered, weighed, and placed in PBS for mixing and centrifugation. After removing the supernatant samples were dissolved in acetic acid with pepsin overnight. The following day the soluble (non-cross-linked) and insoluble (cross-linked) fractions were separated and boiled in 6 M HCl overnight. The resulting lysates were aliquoted and sequentially added to solutions containing chloramine T and Erlich’s reagent. After a 30-min incubation at 58 °C, samples were cooled, centrifuged, and then plated in duplicate for measurement of absorbance at 558 nm. Total collagen content and cross-linking were back calculated using a standard curve and reported as collagen content per powdered mass in μg/mg and percent insoluble collagen.

### 2.8. Statistical analysis

Statistics were performed in GraphPad Prism (version 10.2.3). Sex-based differences were investigated for primary outcomes, but significant differences were not found. Thus, data from both sexes were pooled to increase statistical power. A mixed effects analysis was run in place of an analysis of variance (ANOVA) in cases where some of the mechanical testing data was missing due to mechanical failure in at least one muscle sample. Three-way ANOVAs or mixed effect analyses were run on stress–strain data across genotype, treatment, and strain, and were used on stiffness data for non-decellularized diaphragm strips across strain, genotype, and treatment. Sidak’s multiple comparisons tests and Tukey’s multiple comparisons tests were used to identify pairwise significant differences where appropriate. Two-way ANOVAs or mixed effect analyses were run on stiffness and elastic index data across genotype and treatment with post-hoc Fisher’s LSD tests or Sidak’s multiple comparisons tests where appropriate. Two-way ANOVAs were used for hydroxyproline, collagen solubility, and collagen fiber size/area data for EDL, soleus, and diaphragm muscles across genotype and treatment with post-hoc Sidak’s multiple comparisons tests or Fisher’s least square differences (LSD) tests where appropriate. A two-way ANOVA was also performed on the collagen fiber diameter data for sham-digested WT and D2.*mdx* diaphragm epimysium and perimysium by genotype and ECM layer. Post-hoc Sidak’s multiple comparisons tests were run to identify pairwise differences by ECM layer. T-tests were performed on liver hydroxyproline and collagen solubility data. Simple linear regressions were performed between continuous data sets where relationships were hypothesized. Three-way ANOVAs or mixed effect analyses were run on collagen alignment data across strain, genotype, and treatment with post-hoc Tukey or Sidak’s multiple comparisons tests. A two-way ANOVA was run on the diaphragm epimysium alignment data across genotype and strain with post-hoc Sidak’s multiple comparisons tests. Two-way ANOVAs were run on muscle mass and muscle length data across genotype and treatment with post-hoc Sidak multiple comparisons tests or Fisher’s LSD tests as appropriate.

## 3. Results

### 3.1. Decellularized muscle mechanics

**Fig. 2.**
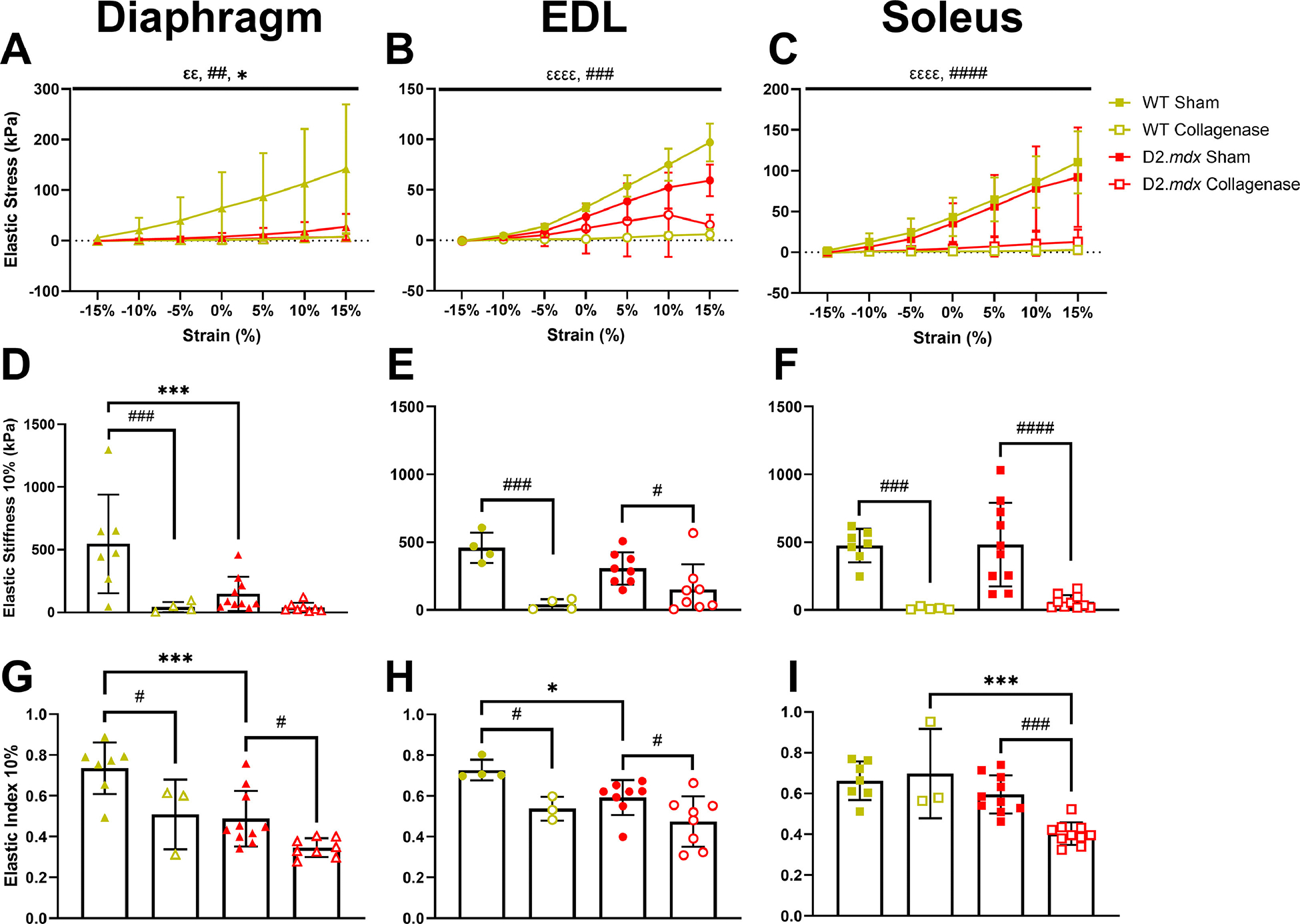
DCM mechanics for diaphragm, EDL and soleus muscles. **(A**-C) Diaphragm, EDL, and soleus DCMs had higher elastic stress with increasing strain and lower elastic stress after collagenase treatment. WT diaphragm DCMs had higher elastic stress than D2.*mdx* DCMs. **(D**-F) Collagenase treatment reduced DCM stiffness. WT diaphragm DCMs were stiffer than D2.*mdx* diaphragm DCMs. **(G**-I) Collagenase reduced elastic index in DCMs. WT diaphragm and EDL DCMs were more elastic than D2.*mdx* counterparts. Data represented by mean ± SD. Flat bars represent significant main effects while brackets represent pairwise differences by post-hoc tests. Significance by strain: *^εε^* =*p* < 0.01, *^εεεε^* =*p* < 0.0001; significance by collagenase treatment: ^#^ *p* < 0.05, ^##^ *p* < 0.01, ^###^ =*p* < 0.001, ^####^ *p* < 0.0001; significance by genotype: ^∗^*p* < 0.05, ^∗∗∗^*p* < 0.001.

**Fig. 3.**
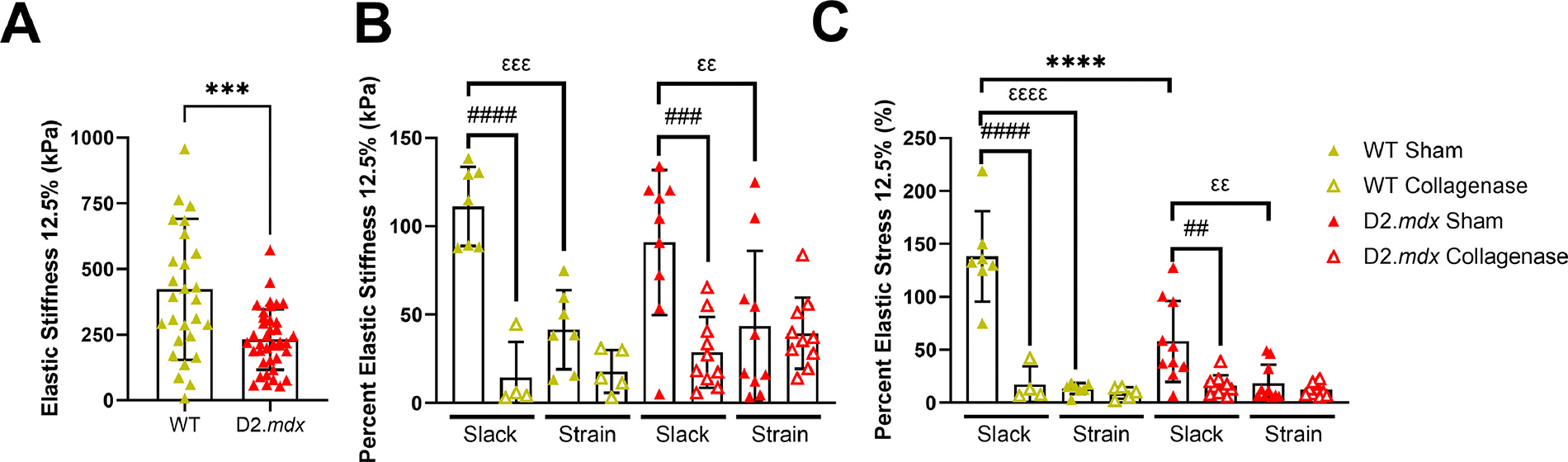
Muscle mechanics of intact diaphragm strips in response to strain and collagenase treatments. (A) Elastic stiffness of WT diaphragm strips was higher than D2.*mdx*. **(B**-C) Holding muscles at strain for 1-hour reduced their elastic stiffness and stress, and collagenase reduced elastic stiffness and stress of muscles. However, muscles held at strain exhibited resistance to collagenase-mediated digestion. D2.*mdx* muscles maintain less stress after a sham digestion than WT. Data represented by mean ± SD. Pairwise differences indicated by brackets. Significance by genotype: ^∗∗∗^*p* < 0.001, ^∗∗∗∗^*p* < 0.0001; significance by strain: *εε p*<0.01, *εεε p*<0.001; significance by collagenase treatment: ^###^ *p* < 0.001, ^####^ *p* < 0.0001.

Although we expected the D2.*mdx* decellularized muscles (DCMs) to have higher passive mechanical properties than wildtype (WT) DCMs, we found the WT muscle mechanics were either similar or greater than that of the D2.*mdx* DCMs. Across all diaphragm, EDL, and soleus DCMs elastic stress increased with strain and decreased with collagenase treatment (Fig. 2A–C). Additionally, the WT diaphragm DCMs had significantly higher elastic stress than the D2.*mdx* diaphragm DCMs (Fig. 2A). Elastic stiffness, the slope of the stress strain curve at 15 % strain, was significantly decreased with collagenase treatment across all DCMs tested (Fig. 2D–F). While there was no genotype difference in elastic stiffness in EDL or soleus DCMs, the WT diaphragm DCM exhibited higher elastic stiffness than D2.*mdx* diaphragm DCM (Fig. 2D). This was paralleled by increased stiffness of intact WT diaphragms compared to D2.*mdx* (Fig. 3A). Elastic index, the ratio of elastic to dynamic stiffness at 15 % strain, was reduced in the D2.*mdx* and collagenase-treated diaphragm DCMs compared to the respective WT and sham-treated diaphragm DCMs (Fig. 2G). Collagenase-digested WT EDL DCMs were less elastic than corresponding sham DCMs (Fig. 2H). Also, collagenase-treated WT soleus DCMs were more elastic than collagenase-treated D2.*mdx* soleus DCMs, and collagenase digestion reduced the elastic index of D2.*mdx* soleus DCMs (Fig. 2I). Dynamic stress and stiffness results demonstrated similar trends as elastic mechanics and are not reported in the figures. Overall, wildtype DCMs were as stiff or stiffer than D2.*mdx* DCMs, and collagenase treatment consistently reduced the stiffness and elastic index of DCMs across all muscles.

### 3.2. Effects of passive tension on collagenase digestion

Based on previous studies we expected passive tension to blunt the effectiveness of collagenase digestion on intact diaphragm muscle strips [52–54]. In line with this hypothesis, we found that collagenase digestion reduced stress and stiffness in slack diaphragm strips to a greater degree than in strained diaphragm strips (Fig. 3B and C). However, we also observed that simply holding the diaphragm strips at strain during a sham treatment reduced their stress and stiffness (Fig. 3B and C). We also observed an effect of genotype on the reduction in stress of slack diaphragm strips following a sham treatment (Fig. 3C). Overall, we find that while holding a muscle at strain will hinder the ability of collagenase to reduce stiffness, the act of holding the muscle at strain leads to a reduction in stiffness and stress independent of collagenase treatment.

### 3.3. Collagen content and cross-linking in decellularized muscles and intact liver

**Fig. 4.**
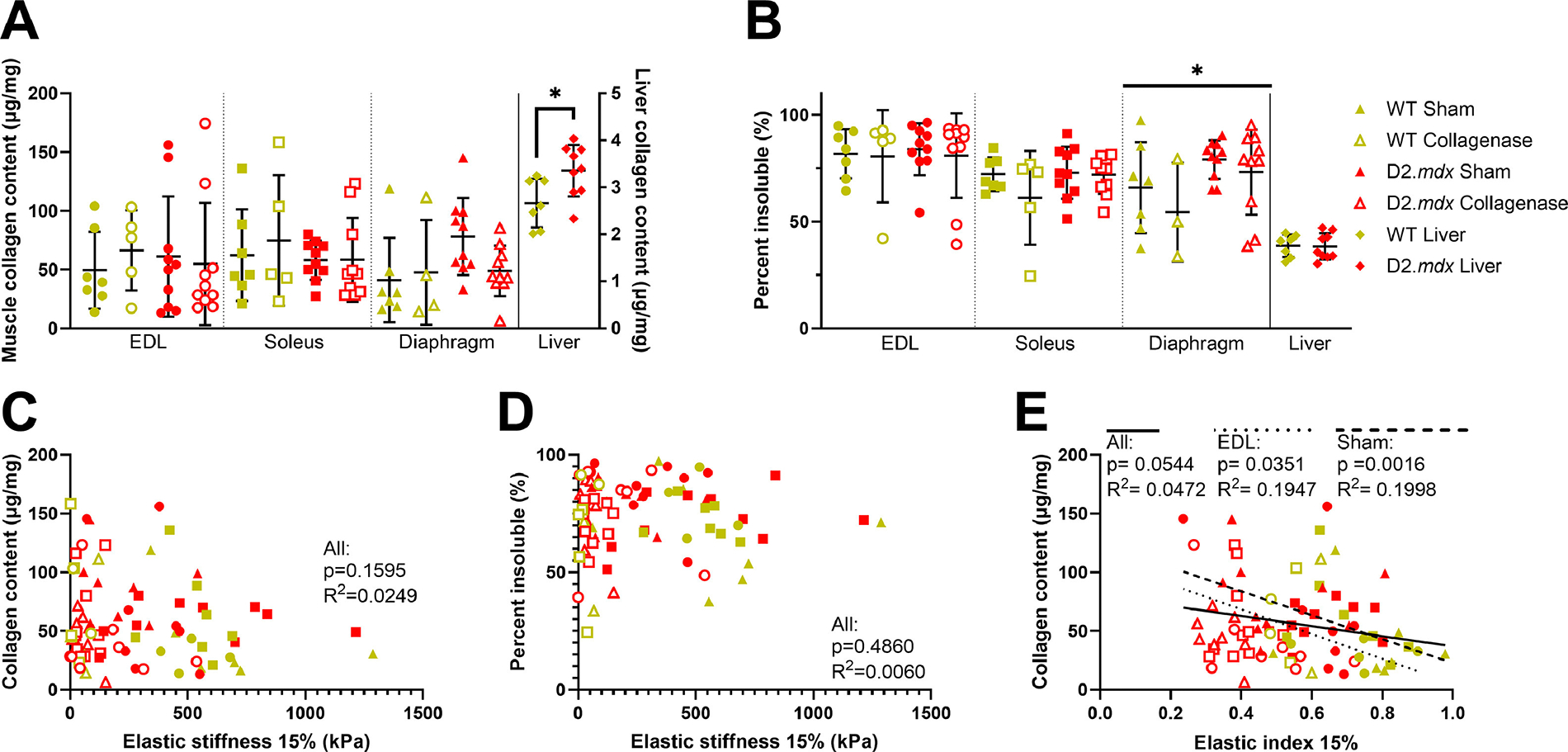
Collagen content and cross-linking in DCMs and liver. (A) Collagenase treatment did not affect the collagen content in WT and D2.*mdx* EDL, soleus, or diaphragm DCMs. Liver collagen content was higher in D2*.mdx* mice than WT mice. (B) Percent of insoluble (cross-linked) collagen was higher in the D2*.mdx* diaphragm DCMs than WT. **(C**-D) There were no significant correlations between collagen content or cross-linking with elastic stiffness. (E) There were significant negative correlations between collagen content and elastic index. Data represented by mean ± SD. Significant main effects represented by flat bars. Brackets represent significant pairwise differences. Significance by genotype: ^∗^*p* < 0.05.

In D2.*mdx* muscles, there is often an increase in collagen content and cross-linking compared to WT [10,35,36,38]. However, we did not observe a significant difference in collagen content between WT and D2.*mdx* DCMs, but we did observe an increase in liver collagen content in the D2.*mdx* mice compared to WT (Fig. 4A). We also found that D2.*mdx* diaphragm DCMs had higher percentages of insoluble (cross-linked) collagen than WT diaphragm DCMs (Fig. 4B). Interestingly, there was no effect of collagenase treatment on collagen content or cross-linking in any DCM tested (Fig. 4A and B). Neither collagen content nor percent of insoluble collagen were significantly correlated to elastic stiffness across muscles (Fig. 4C and D). Nevertheless, a smaller amount of collagen content was significantly correlated to a lower elastic index in the EDL, sham muscles, and across all groups (Fig. 4E). Altogether, D2.*mdx* diaphragm DCMs had a higher degree of cross-linked collagen than WT, but neither collagen content nor cross-linking were correlated to elastic stiffness.

### 3.4. Collagen fiber size and area

**Fig. 5.**
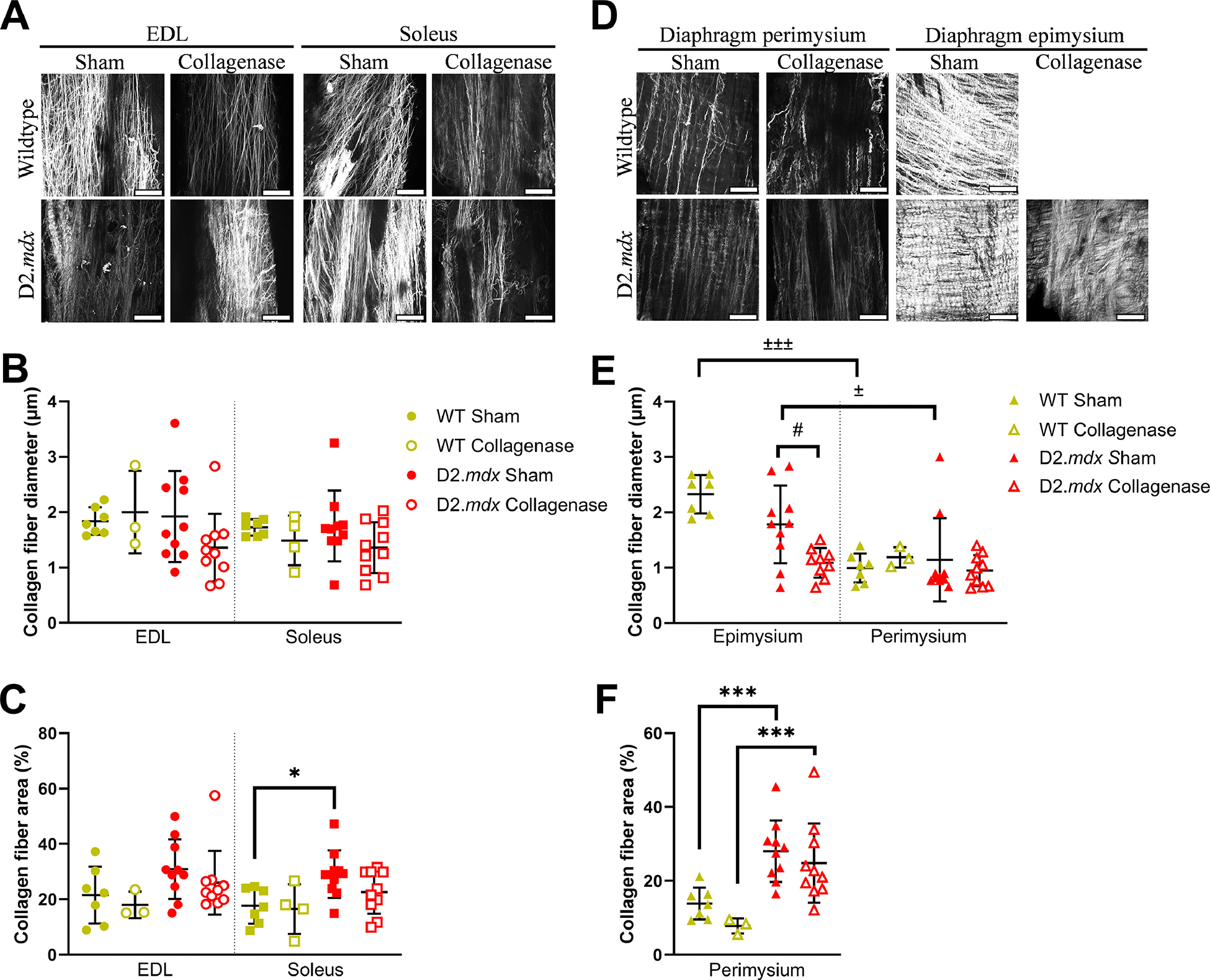
Collagen fiber diameter and area. (A) Representative SHG images of WT and D2.*mdx* EDL and soleus muscles with or without collagenase treatment. (B) Collagen fiber diameter was not significantly different by genotype or collagenase treatment in EDL and soleus muscles. (C) Collagen fiber area was significantly higher in D2.*mdx* soleus muscles than WT. (D) Representative SHG images of WT and D2.*mdx* diaphragm DCMs with and without collagenase treatment. (E) Collagen fiber diameter was significantly higher in diaphragm epimysium than perimysium. Collagen fiber diameter in D2.*mdx* epimysium was decreased with collagenase digestion. (F) Collagen fiber area was significantly higher in D2.*mdx* diaphragm perimysium and WT. Scale bar=100 μm. Data represented by mean ± SD. Significant pairwise differences by ECM layer: ^±^*p* < 0.05, ^±±±^*p* < 0.001; genotype: ^∗^*p* < 0.05, ^∗∗∗^*p* < 0.001; and collagenase treatment: ^#^ *p* < 0.05.

Using SHG imaging we were able to calculate collagen fiber architectural properties including collagen fiber size and area fraction. (Fig. 5A,D). We found no significant differences in EDL or soleus collagen fiber diameter by genotype or collagenase treatment (Fig. 5B). However, we did observe that D2.*mdx* collagen fibers within the soleus DCM occupied a greater area than in the WT DCM (Fig. 5C). After examining the SHG images for all the diaphragm DCMs, we observed that the collagen fiber architecture at the surface (epimysium) and within the muscle belly (perimysium) were remarkably different (Fig. 5D). Thus, for images of the diaphragm DCMs that contained SHG signal from the surface (epimysium) in addition to signal within the muscle belly (perimysium), we analyzed the architecture of each layer separately (Figs. 5D, 7A). We found that collagen fibers within the sham-digested epimysium were larger than those in the perimysium layer (Fig. 5E). We also saw that collagenase digestion reduced the diameter of collagen fibers within the D2.*mdx* epimysium (Fig. 5E). Lastly, we observed a robust increase in the collagen fiber area fraction within the D2.*mdx* diaphragm perimysium compared to WT (Fig. 5F). These data show that the D2.*mdx* muscle ECM tends to have greater area occupied by collagen fibers, and the epimysium of the diaphragm has larger collagen fibers than the perimysium.

### 3.5. Strain-dependent dynamic re-alignment of collagen fibers

**Fig. 6.**
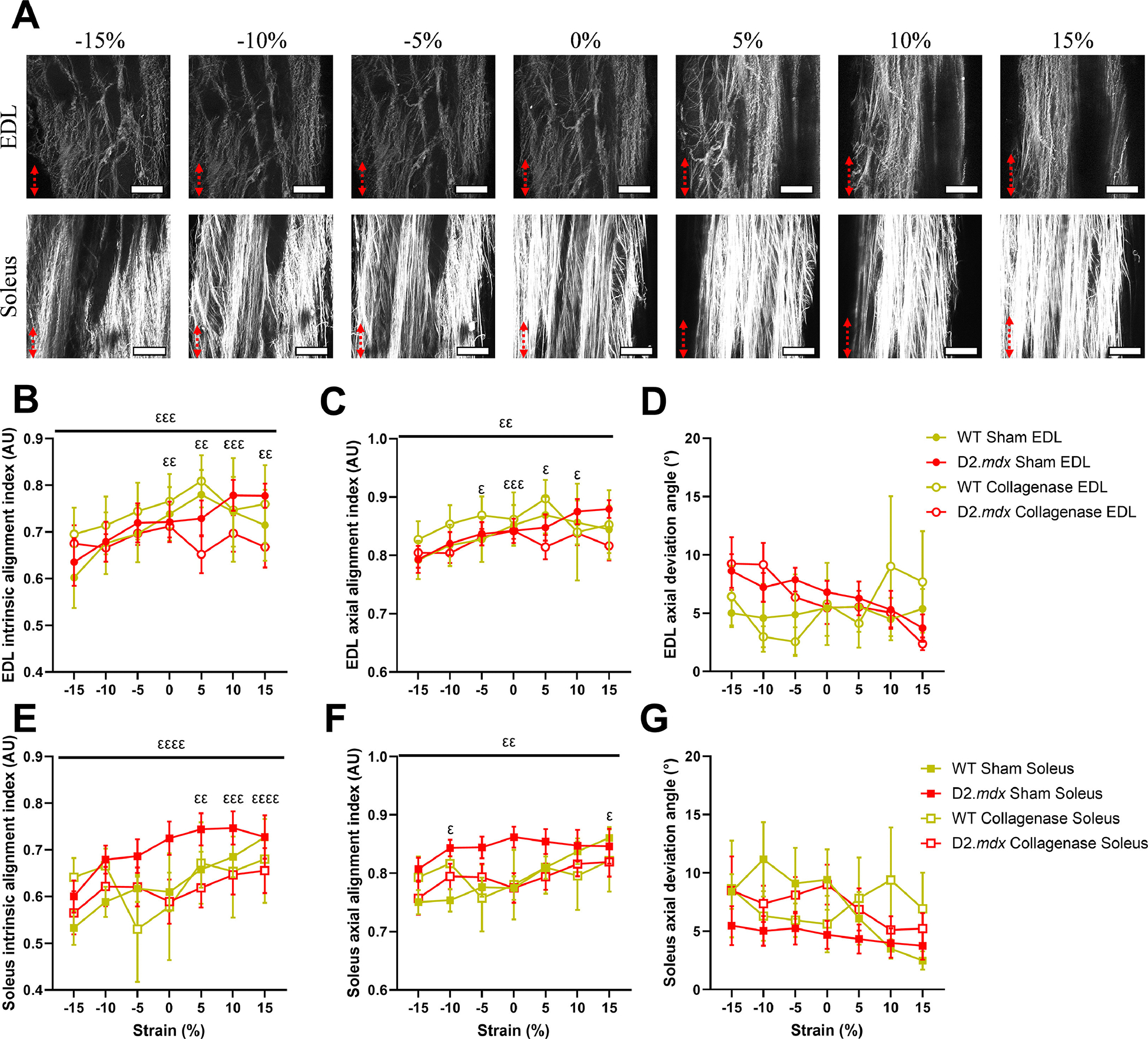
Strain-dependent collagen fiber re-alignment in EDL and soleus DCMs. (A) Representative SHG image series across a range of strains for sham-treated EDL and soleus DCMs show strain-dependent re-alignment of collagen fibers. Red arrows indicate direction and intensity of strain. (B) Collagen fibers within EDL DCMs significantly increased their intrinsic alignment with strain. (C) Axial alignment index, a measure of collagen fiber alignment compared to the axis of strain, increased with strain in EDL DCMs. (D) Axial deviation angle, the difference between the mean collagen angle and angle of strain (90°), did not significantly change with strain in EDL DCMs. (E) Collagen fibers within soleus DCMs significantly increased their intrinsic alignment with strain. (F) Axial alignment index of collagen fibers within soleus DCMs increased with strain. (G) Axial deviation angle did not change with strain in soleus DCMs. Scale bar=100 μm. Data represented by mean ± SEM. Significant main effects represented by flat bars. Significant pairwise comparisons from post-hoc tests represented by symbols above data points. All pairwise comparisons by strain are relative to −15 % strain. Significance by strain: *^εε^ p*<0.01, *^εεε^ p*<0.001, *^εεεε^ p*<0.0001.

By imaging the decellularized muscles over a range of strains, we were able to demonstrate the strain-dependent changes in collagen fiber alignment in WT and D2.*mdx* DCMs (Fig. 1). In EDL and soleus DCMs, we did not observe differences in collagen fiber alignment by genotype or collagenase treatment (Fig. 6A–G). However, we found robust increases in collagen fiber intrinsic and axial alignment in both EDL and soleus DCMs with increasing muscle strain (Fig. 6B and C, E and F). Both intrinsic alignment index (collagen alignment relative to the mean collagen angle) and axial alignment index (collagen alignment relative to the angle/axis of strain) increased with strain in EDL and soleus DCMs (Fig. B-C, E-F). However, we did not observe a strain-dependent change in axial deviation angle, the difference between the mean collagen angle and axis of strain, in EDL or soleus DCMs (Fig. D,G). Thus, in the limb decellularized muscles collagen fibers became more collectively aligned and oriented along the axis of strain with increasing strain. However, the angular deviation between the collagen fibers and the axis of strain did not significantly change as the DCM was strained.

**Fig. 7.**
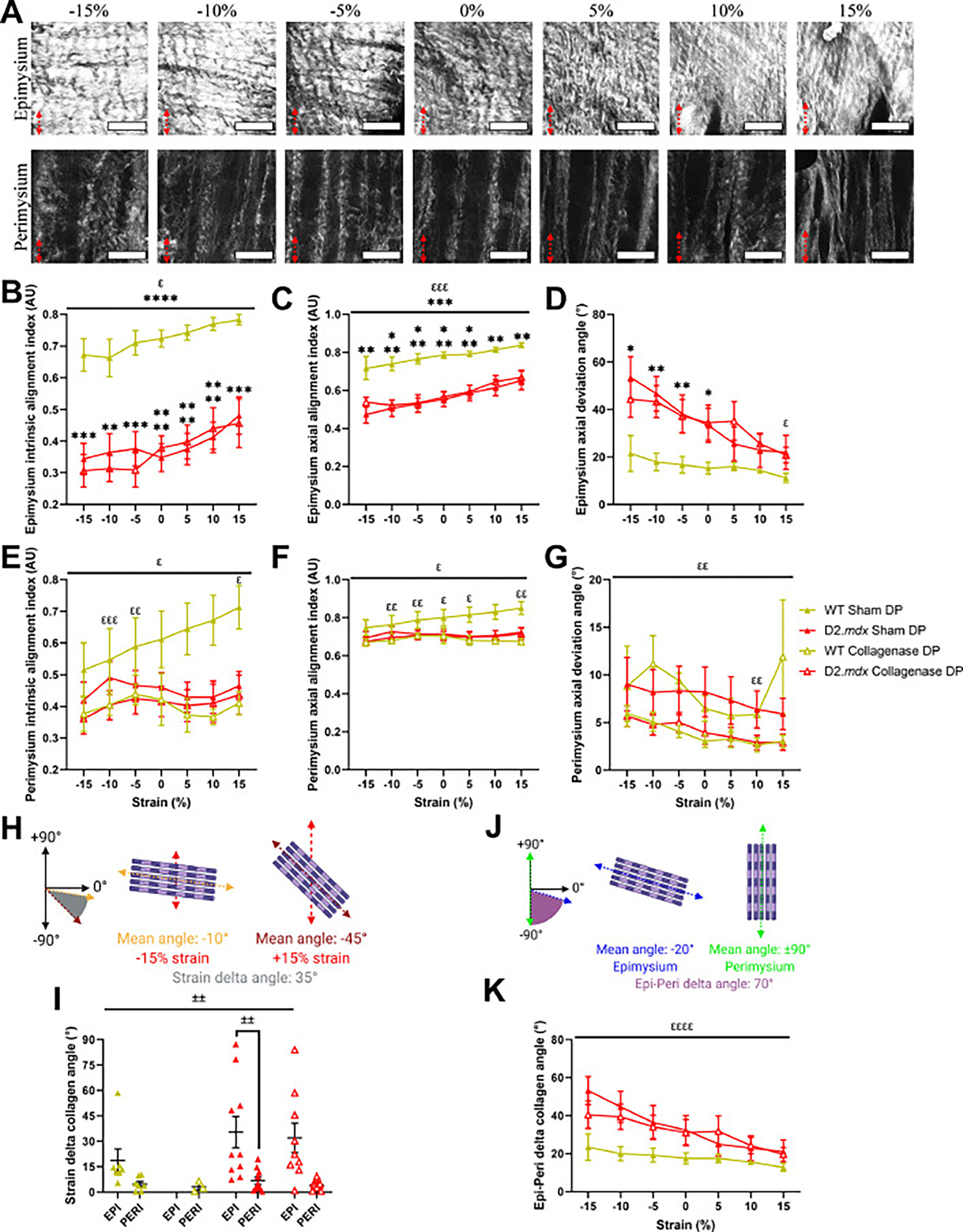
Strain- and layer-dependent dynamic changes in diaphragm DCM collagen fiber alignment. (A) Representative SHG images series across a range of strains of shamtreated diaphragm (DP) DCM epimysium and perimysium show dynamic changes in collagen alignment. Red arrows indicate direction and intensity of strain. Perimysium and epimysium were analyzed separately due to visibly different collagen architecture. **(B**-C) Collagen fiber intrinsic and axial alignment increased with strain in epimysium of diaphragm DCMs. WT diaphragm DCM epimysium collagen was more aligned than D2.*mdx*. (D) Epimysial axial deviation angle decreased with strain. D2.*mdx* epimysial axial deviation angle was significantly higher than WT from −15 % to 0 % strain and decreased from −15 % to 15 %. **(E**-F) Diaphragm perimysial collagen fibers changed their intrinsic and axial alignment with strain. WT perimysial collagen significantly increased intrinsic alignment from −15 % to 15 % strain. (G) Perimysium axial deviation angle decreased with strain. (H) Schematic for measurement of strain delta angle (the difference between the mean collagen angle at −15 % and 15 % strain). (I) The strain delta collagen angle was higher in the epimysium (EPI) compared to perimysium (PERI) for sham treated diaphragm DCMs. (J) Schematic for measurement of Epi-Peri delta angle (the difference between the mean collagen angle of epimysium and perimysium at a given strain). (K) The Epi-Peri delta collagen angle decreased with strain. Scale bar=100 μm. Data represented by mean ± SEM. Significant main effects represented by flat bars. Significant post-hoc differences are represented by brackets or symbols above data points. All pairwise comparisons by strain are relative to −15 % strain. Significance by strain: *^ε^* p < 0.05, *^εε^* p < 0.01, *^εεε^* p < 0.001, *^εεεε^* p < 0.0001; significance by genotype: ^∗^*p* < 0.05, ^∗∗^*p* < 0.01, ^∗∗∗^*p* < 0.001, ^∗∗∗∗^*p* < 0.0001; significance by ECM layer: ^±±^*p* < 0.01.

As previously mentioned, the collagen architecture of the decellularized diaphragm epimysium and perimysium were remarkably different, so they were analyzed separately (Fig. 7A). While we did not observe the epimysium in any image stacks from the WT collagenase digested diaphragm DCMs, we did see that the epimysial collagen fibers increased their intrinsic and axial alignment with strain, and the WT diaphragm DCM had higher alignment across strains compared to the D2.*mdx* diaphragm DCM (Fig. 7B and C). Further, we observed that as diaphragm DCMs were strained, epimysial collagen fibers changed their angle to be closer to the axis of strain (Fig. 7D). This effect was most pronounced in the D2.*mdx* diaphragm DCM, which had a significant reduction in axial deviation angle at 15 % strain compared to −15 % (Fig. 7D). We found that the perimysial collagen fibers within the WT diaphragm DCM linearly increased intrinsic alignment with increasing muscle strain, while the other diaphragm DCMs displayed less consistent changes in alignment with strain (Fig. 7E), although this difference was less pronounced in the axial alignment of collagen fibers (Fig. 7F). Perimysial collagen fibers also tended to reduce their axial deviation angle with increased DCM strain (Fig. 7G).

Since at low strain the collagen fibers in the epimysium started at a greater axial deviation angle compared to the fibers in the perimysium, we predicted that with increasing strain epimysial collagen fibers would exhibit a greater change in collagen angle in response to strain compared to perimysial collagen. Thus, we calculated the change in collagen angle from the lowest to the highest strain (strain delta angle) in epimysium and perimysium to observe whether epimysial collagen fibers changed their mean angle during strain to a greater degree than perimysial collagen fibers. (Fig. 7H). In line with this prediction, we observed that the WT and D2.*mdx* epimysial collagen fibers experienced a greater change in mean collagen angle across the range of strains than their respective perimysial collagen fibers (Fig. 7I). Additionally, we expected that the difference in mean angle between the epimysial and perimysial collagen fibers (Epi-Peri delta collagen angle) would become smaller with increasing strain due to concomitant re-alignment of the collagen in both layers towards the axis of strain (Fig. 7J). Evidently, we observed that the difference in mean angle between decellularized diaphragm epimysium and perimysium significantly decreased with increasing strain, showing that the two layers of the ECM tend to re-align in the same direction as the muscle was strained (Fig. 7K). Overall, we found that as the muscle ECM is strained collagen fibers dynamically re-align along the axis of strain regardless of their initial orientation.

### 3.6. Mechanical significance of collagen fiber alignment

**Fig. 8.**
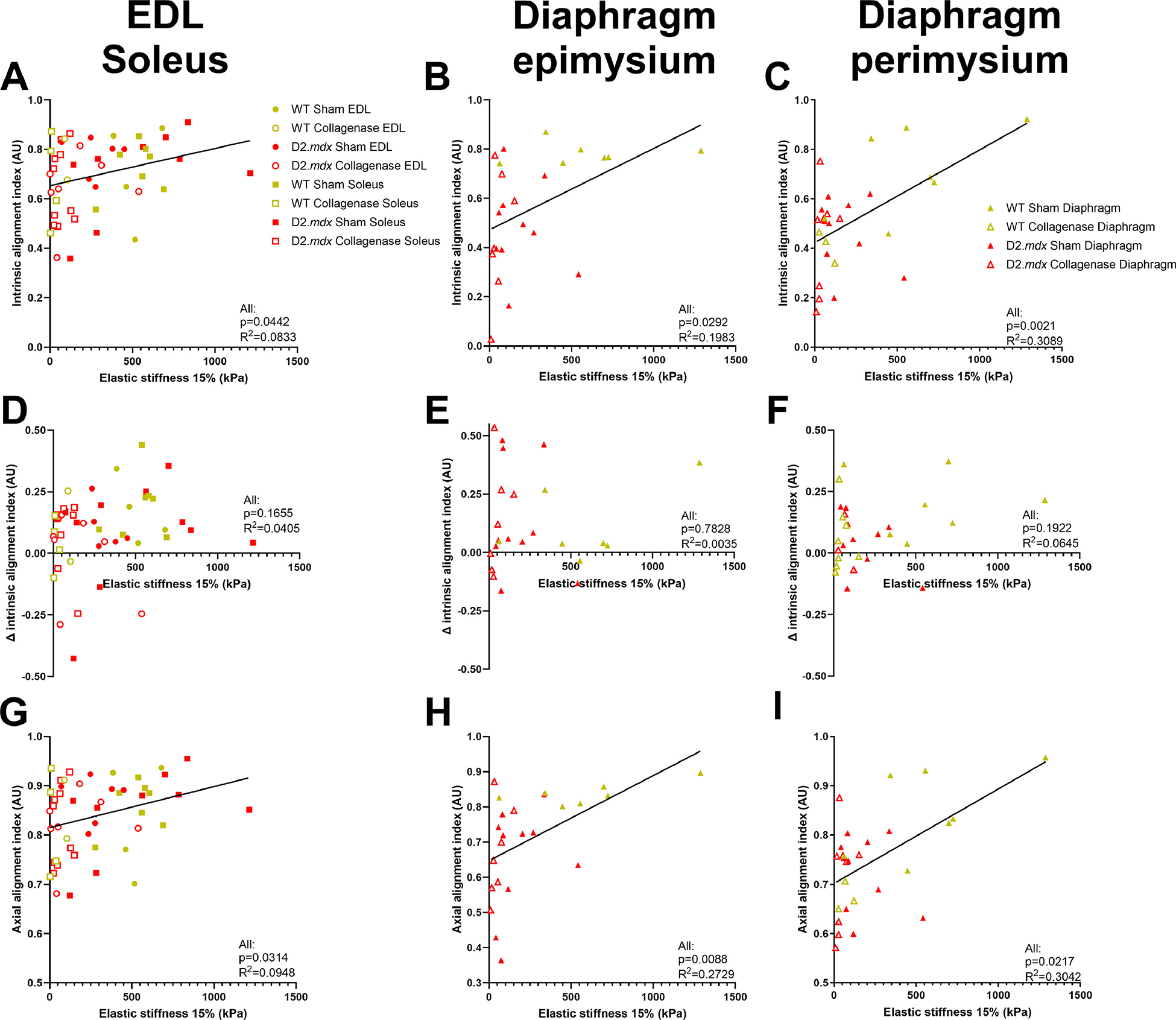
Relationships between collagen alignment and elastic stiffness in DCMs. **(A**-C) There were significant weak positive correlations between collagen alignment and elastic stiffness at 15 % strain. **(D**-F) The delta in collagen alignment up to 15 % strain was not significantly related to elastic stiffness. **(G**-I) Strain alignment index was significantly correlated to elastic stiffness at 15 % strain.

**Fig. 9.**
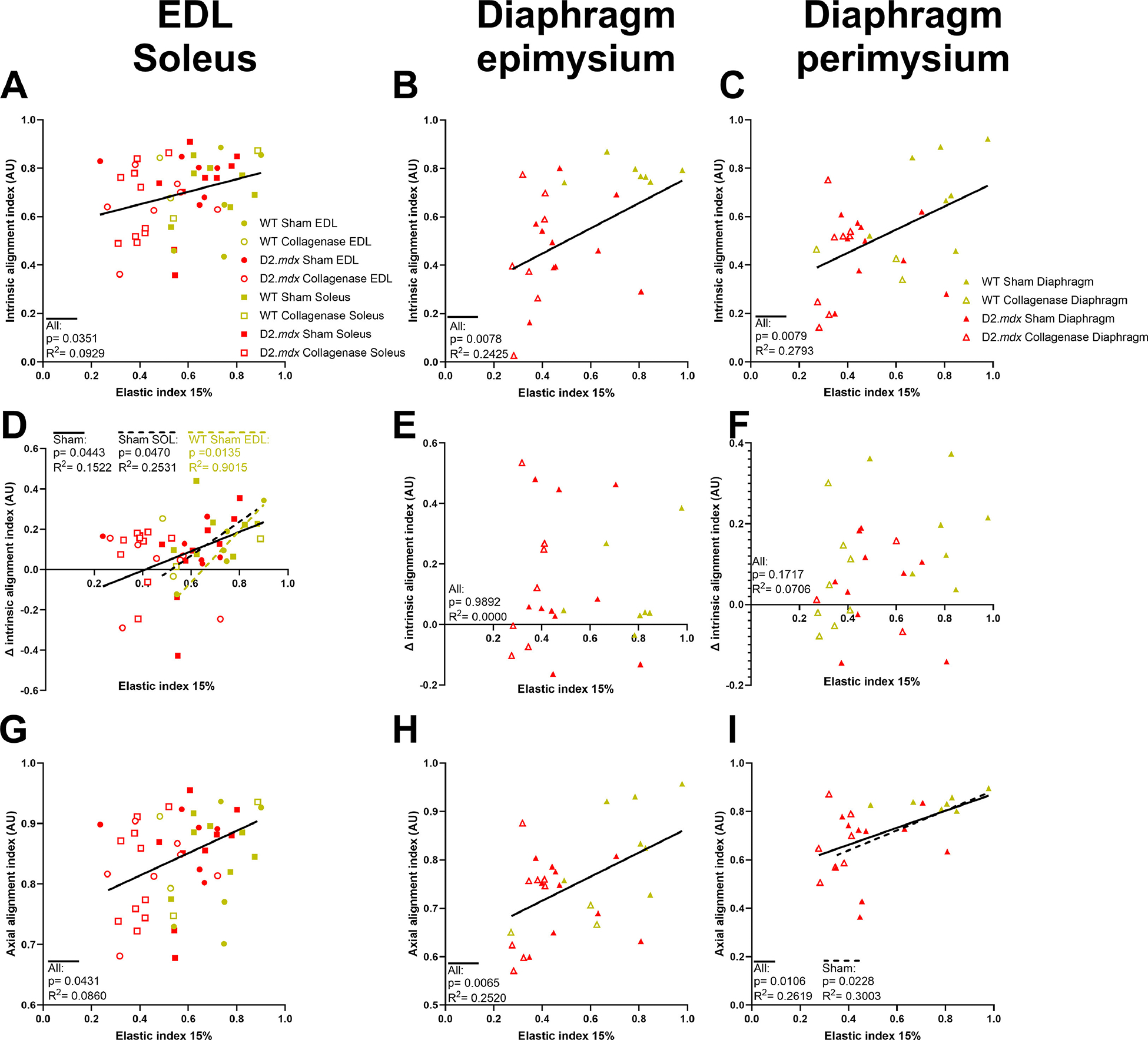
Relationships between collagen alignment and elastic index of DCMs. **(A**-C) Intrinsic alignment index was significantly correlated to elastic index at 15 % strain in EDL, soleus, and diaphragm DCMs. **(D**-F) Delta intrinsic alignment index up to 15 % strain correlated to elastic index in sham EDL and soleus DCMs. **(G**-I) Axial alignment index was significantly correlated with elastic index in all DCMs tested.

Lastly, we compared the relationship between dynamic collagen alignment and DCM elastic stiffness. We found that collagen alignment in the EDL and soleus muscles, as well as diaphragm perimysial and epimysial alignment were correlated to increased elastic stiffness (Fig. 8A–C). However, we did not find significant correlations between these parameters for any individual muscle group or genotype. However, we did not observe a significant correlation between the change in alignment and elastic stiffness (Fig. 8D–F). In parallel with alignment index, strain alignment index was significantly correlated to stiffness in the EDL, soleus, and diaphragm (Fig. 8G–I). In addition to stiffness, we investigated correlations between elastic index and collagen alignment, and found that the alignment index and strain alignment index were correlated to elastic index in all DCMs tested (Fig. 9A–C, G–I). We also observed that delta collagen alignment index was associated with elastic index in sham EDL and soleus DCMs, but not in the diaphragm (Fig. 9D–F). In short, we found that collagen fiber alignment was weakly related to DCM mechanical properties.

## 4. Discussion

### 4.1. Middle-aged dystrophic diaphragm ECM is less stiff than WT

In general, dystrophic muscles from the D2.*mdx* mouse tend to be stiffer than WT muscles [10,35,36]. Previous reports have demonstrated that changes across multiple aspects of the muscle collagen matrix including collagen content, cross-linking, fiber density, and alignment are correlated to increased stiffness in dystrophic muscle [10,35–38,55]. Further, the D2.*mdx* diaphragm tends to be stiffer than its WT counterpart and compared to limb muscles in the D2.*mdx* mouse [10,36]. However, most studies that have investigated the skeletal muscle pathology of D2.*mdx* mice assessed the phenotype in young and adult mice (~8–35 weeks old) rather than middle-aged mice (>47 weeks old). Surprisingly, our data show that the middle-aged D2.*mdx* diaphragm DCM had approximately one-quarter of the elastic stiffness of the matched WT, and the intact middle-aged D2.*mdx* diaphragm was about half as stiff than the WT (Figs. 2D, 3A). We would expect a drop in ECM stiffness to be related to a decrease in collagen content, crosslinks, and alignment. While we found that the D2.*mdx* diaphragm had more cross-links and perimysial collagen fiber area than the WT diaphragm (Fig. 4A and B, Fig. 5F), the dystrophic diaphragm had reduced collagen alignment, particularly in the epimysium, compared to the WT (Fig. 7B and C). However, we did not observe a strong relationship between epimysial alignment and stiffness that would explain the genotype-based difference in stiffness between diaphragms (Fig. 8B,E). Since the collagen architecture of the WT and D2.*mdx* diaphragms did not fully explain the difference in stiffness, we infer that age-related changes to the ECM such as proteoglycan content, composition of various collagens, and types of cross-links may be larger contributors to the lower stiffness of the D2.*mdx* diaphragm ECM. Previous reports support a general increase in the synthesis of ECM components within muscle during aging including collagens 1, 4, and 6; small-leucine rich proteoglycans (SLRPs) decorin, biglycan, lumican, and asporin; and other components associated with the ECM including fibrillin-1, laminins, and integrins [56,57]. While collagen has an important load-bearing role in muscle ECM, other matrix components contribute to mechanical properties. Glycosaminoglycan (GAG) chains on proteoglycans can contribute to hydrostatic pressure within the ECM, which can regulate mechanical properties [58,59]. The function of SLRPs is also important to the proper formation and organization of the collagen matrix [60–62]. Thus, the content and functions of proteoglycans are integral to the regulation of ECM mechanical properties. Minor components like fibrillin-1, laminin, and integrins form microfibril networks [63] or contribute to the stability of the muscle-matrix connection at the sarcolemma [64,65]. Collagen cross-links are robustly increased with aging [66,67], specifically advanced glycation end-products (AGE) [68–70], although we did not see a clear relationship between cross-link content and elastic stiffness. While there is a plethora of ECM components that increase in content with aging, determining how each would individually alter muscle stiffness is more difficult. Also, it is possible that since the D2.*mdx* diaphragm is thicker than the WT, that differences in the mass of the diaphragm strips underpredict the *in vivo* passive mechanical properties of the D2.*mdx* diaphragm compared to WT. In any case, these findings of decreased ECM stiffness in dystrophic muscle compared to WT are surprising and warrant future investigation to determine their structural basis.

### 4.2. Strain-mediated resistance to collagenase digestion

Previous studies suggest that collagen fibers under higher tension are more resistant to enzymatic degradation by collagenase [52–54]. We tested this concept in muscle by measuring the elastic stiffness of diaphragm strips before and after a sham or collagenase treatment while at slack length or 12.5 % strain. As expected, we found that the collagenase-associated reduction in stiffness was blunted by holding a muscle under tension (Fig. 3B). We also observed that holding a muscle at tension lowered stiffness regardless of collagenase treatment (Fig. 3B). Because the sham or collagenase treatment was an hour long, it is possible that stress-relaxation effects that occurred over the course of the 1-h treatment were not reversed by the time post-treatment mechanical testing began. Based on this assumption, when muscles were held at a slack length for one hour, they did not experience stress relaxation and exhibited comparable mechanical properties to what was measured prior to the 1-h treatment. However, the muscles that were held at tension for one hour lost about half or more of their previous stiffness due to the stress-relaxation of collagen and elastic elements in the muscle that was not undone before post-treatment mechanical testing. Interestingly, the collagenase treatment did not reduce the stiffness of muscles held at tension while it severely reduced the stiffness of muscles held at slack length. Put together, even if the collagen matrix underwent extensive stress-relaxation during the 1-h treatment under tension, the collagen matrix was still resistant to the effects of the collagenase compared to the muscles digested at slack length. While quantifying the degree of stress-relaxation in isolated skeletal muscles is outside the scope of this study, we anticipate this could be achieved by holding muscles at strain for different time periods from several minutes to an hour and then measuring their passive mechanical properties immediately after and several minutes following the held strain. Some studies have already looked at stress-relaxation in muscle and could be used as a guide for future research [71,72]. Further investigation is needed to elucidate the impact of stress-relaxation on muscle stiffness following a held strain, and the proportionality between the degree of muscle strain and level of collagenase resistance.

### 4.3. *Increased collagen content in D2.*mdx *liver*

While it is well documented that the muscles in the D2.*mdx* mouse exhibit progressive fibrosis that is more severe than other genetic backgrounds [38,73], it is not known if other organs become fibrotic in the mouse. There is evidence that the DBA/2J background has increased transforming growth factor-*β* (TGF*β*) signaling due to genetic variants in latent TGF*β*-binding-protein 4 (LTBP4) which could lead to upregulated expression of pro-fibrotic transcriptional pathways [74,75]. While we did not test another genetic background of mice in this study, we performed the hydroxyproline and collagen solubility assay on liver tissue from WT and D2.*mdx* mice to see if collagen content and cross-links were altered across genotypes. We found that there was more collagen in the D2.*mdx* liver than in the WT, but no difference in cross-linking (Fig. 4D). Compared to typical total collagen values in other mice, we find that collagen content in the DBA/2J mice is 6–25 fold higher than mice on other genetic backgrounds [76–78]. This would suggest that the DBA/2J background has an exponentially more severe pro-fibrotic transcriptional program than other genetic backgrounds. While the hydroxyproline assay is performed similarly between studies, differences in protocols could lead to altered collagen content measurements. Future study could elucidate what other factors lead to this massive increase in liver collagen content in DBA/2J mice.

### 4.4. Dynamic collagen fiber architecture in epimysium and perimysium

It has long been hypothesized that the muscle collagen matrix responds to changes in muscle length with changes in fibril orientation [24–26,28,29]. While there have been many studies that show a difference in fibrillar collagen alignment in muscles under different strains, few if any have performed imaging on the same muscle across a range of strains. Our study is unique in that we performed SHG microscopy on DCMs across a range of strains to visualize and quantify changes in collagen fiber alignment as muscles are stretched. We found that collagen fibers increased their intrinsic and axial alignment with increasing muscle strain (Fig. 6B and C, E and F; 7B–G). It is understood that collagen fibrils *in vitro* change their orientation in response to loading [17,79–81], thus, as muscle lengthens *in vivo* the collagen fibers within the ECM incur a tensile load that leads them to increase their alignment along the axis of load or strain. Given the collagenase treatment robustly reduced the stiffness of all muscles tested, we expected the collagen fibers to incur less load and change their alignment to a lesser degree. Surprisingly, we did not observe an effect of collagenase on the re-alignment of collagen fibers during muscle stretching (Figs. 6B–G, 7B–G). From this finding, we interpret that although digesting collagen fibers with collagenase reduces their mechanical properties, the collagen fibers still alter their orientation in response to a change in muscle length.

Another interesting finding was the difference between the epimysium and perimysium architecture within the diaphragm. While we did not detect a difference in collagen organization in the limb muscles between the surface and inner muscle belly, the diaphragm clearly displayed architectural differences such that the collagen fibers at the surface were thicker and oriented farther away from the muscle long axis, while the perimysial collagen fibers were thinner and oriented more parallel to the direction of muscle fibers (Fig. 5D and E; Fig. 6C and D, F and G; Fig. 7C and D, F and G, I). While the perimysial collagen did not exhibit large changes in the mean collagen angle as the diaphragm strip was strained, we found that the epimysial collagen had much larger changes in mean collagen angle towards the axis of strain as the DCM incurred more strain (Fig. 7D,G,I). Interestingly, we observed that the D2.*mdx* and collagenase-digested diaphragm perimysium did not exhibit clear strain-dependent increases in collagen alignment (Fig. 7E, and F). This may be due to a compromise in loading between epimysial and perimysial collagen fibers in the D2.*mdx* muscles or with collagenase digestion. The WT epimysium had a much higher degree of intrinsic and axial collagen alignment than the D2.*mdx* (Fig. 7B and C). One potential explanation for this difference could be changes in collagen fiber crimp between WT and D2.*mdx* that could lower the measured alignment in the dystrophic epimysium, however, we did not measure collagen crimp in this study. Taken with the observation that the difference in mean collagen angle between the diaphragm perimysium and epimysium decreased with increasing muscle strain (Fig. 7K), and the strain-dependent increase in axial collagen alignment in most of the DCMs tested (Figs. 6C,F; 7C), we conclude that collagen fibers tend to re-align along the axis of stretch regardless of their initial orientation.

### 4.5. Biomechanical implications of dynamic collagen re-alignment

The muscle ECM is regionally organized such that muscle strain induces changes in collagen fiber angle and straightness depending on the initial orientation of collagen and direction of tensile loading [24,26,58,82]. Based on the mechanical properties of collagen fibrils *in vitro*, changes in collagen fiber angle should correspond to smaller changes in passive mechanics compared to changes in collagen fiber straightness [17]. This appears to be the case in the endomysium, which contains a fibrous layer of collagens that are oriented circumferentially around the muscle fibers [26,29]. Endomysial collagen fibers primarily alter their fiber angle rather than their straightness in response to changes in muscle length [25,26]. In parallel, the endomysium does not seem to exhibit significant mechanical properties on the level of the whole muscle compared to the perimysium and epimysium, which exhibit changes in collagen fiber angle and straightness in response to muscle strain [24,25,28]. However, in our study we did observe weak, but significant, relationships between measures of collagen alignment and mechanical properties. This could be due to a smaller portion of collagen-based mechanics coming from the alignment of fibers and more coming from the molecular sliding that was not measured in this study. Much of the non-linearity of collagen tensile mechanics is due to intermolecular sliding within fibrils [17,22], which could explain how a large increase in passive DCM mechanics was not highly correlated to alignment alone. However, intermolecular sliding in collagen fibrils occurs following the re-alignment of collagen, so a higher starting alignment of the muscle collagen matrix could potentially prime fibers for molecular sliding to occur at a lower strain. For this reason, it is important to continue studying the significance behind collagen fiber organization in skeletal muscle to fully understand how disease remodels the ECM in a way that alters mechanical properties. Further, given the regional differences in collagen architecture throughout the muscle, it is worth investigating how muscle resident cells respond to changes in collagen angle and collagen straightness, as those architectural properties are altered in disease.

## 5. Limitations

Our study was limited in the time separation of the mechanical testing and SHG imaging techniques. At the time of this study, the available SHG microscope was not compatible with concurrent mechanical testing due to space limitations on the microscope stage. However, future studies should consider technical strategies to combine muscle mechanics with SHG. Other imaging modalities are also compatible with concurrent muscle mechanical testing, including confocal and widefield fluorescence and brightfield imaging, however, SHG is unique in that no fixatives or additional stains or fluorophores are necessary to visualize collagen. While we did not originally intend to image the diaphragm epimysium for comparison of epimysial and perimysial architecture, we were able to make this comparison due to the signal from the epimysium in the majority of our image set. However, we found that none of our SHG images from the collagenase treated WT diaphragm DCMs contained signal from the epimysium. Because of this, we did not report epimysial architecture data from this group, limiting the breadth of our analysis. We elected to use DCMs for the majority of this study in order to expand the depth to which we could penetrate the sample for SHG imaging, and to more closely connect changes in collagen architecture to ECM mechanics. We acknowledge that the mechanical properties of DCMs are different from whole muscle, and that our decellularization protocol may induce some swelling of the sample [83]. However, our decellularization technique was free of detergents and promotes high conservation of ECM components and organization [10,11,83]. Finally, our metric for collagen alignment index does not distinguish between collagen angular alignment and collagen straightness. However, we still showed robust patterns of increased collagen alignment with muscle strain, and we reported results on the mean collagen angle in the diaphragm, where there were visible changes in collagen angle between the epimysium and perimysium.

## 6. Conclusions

Through integration of whole muscle mechanical testing and SHG microscopy this is the first study to robustly demonstrate dynamic re-alignment of perimysial collagen with muscle strain. The relationship between strain and collagen alignment was even more profound in the epimysium of the diaphragm with its distinct architecture. There were significant relationships between muscle tissue stiffness with both axial and intrinsic collagen alignment, and the change in intrinsic alignment as decellularized muscles incurred strain. However, these relationships were weak, implicating other factors of ECM architecture as determinants of stiffness. This was highlighted by the surprising result that the highly fibrotic D2.*mdx* diaphragm was significantly less stiff than the WT. Further, collagenase treatment of DCMs dramatically reduced stiffness, although less so when the tissue was under strain. Despite the change in stiffness, collagenase did not produce discernable changes in visualization of collagen fibers and their alignment. Together these results show that the muscle ECM undergoes dynamic changes in collagen architecture in response to strain, but collagen alignment alone does not completely explain the impact of ECM architecture on matrix stiffness.

## References

[1] Gordon AM, Huxley AF, Julian FJ, The variation in isometric tension with sarcomere length in vertebrate muscle fibres, J. Physiol. 184 (1966) 170–192, doi:10.1113/jphysiol.1966.sp007909. 5921536 PMC1357553

[2] Hill DK, Tension due to interaction between the sliding filaments in resting striated muscle. the effect of stimulation, J. Physiol. (Lond.) 199 (1968) 637–684, doi:10.1113/JPHYSIOL.1968.SP008672. 5710425 PMC1365364

[3] Labeit S, Kolmerer B, Titins: giant proteins in charge of muscle ultrastructure and elasticity, Science 270 (1995) 293–296, doi:10.1126/SCIENCE.270.5234.293. 7569978

[4] Freundt JK, Linke WA, Titin as a force-generating muscle protein under regulatory control, J. Appl. Physiol. 126 (2019) 1474–1482, doi:10.1152/JAPPLPHYSIOL.00865.2018. 30521425

[5] Meyer GA, Lieber RL, Elucidation of extracellular matrix mechanics from muscle fibers and fiber bundles, J. Biomech. 44 (2011) 771–773, doi:10.1016/J.JBIOMECH.2010.10.044. 21092966 PMC3042517

[6] Ward SR, Winters TM, O’Connor SM, Lieber RL, Non-linear scaling of passive mechanical properties in fibers, bundles, fascicles and whole rabbit muscles, Front. Physiol. 11 (2020) 211, doi:10.3389/fphys.2020.00211. 32265730 PMC7098999

[7] Prado LG, Makarenko I, Andresen C, Krüger M, Opitz CA, Linke WA, Isoform diversity of giant proteins in relation to passive and active contractile properties of rabbit skeletal muscles, J. Gen. Physiol. 126 (2005) 461–480, doi:10.1085/JGP.200509364. 16230467 PMC2266601

[8] Marcucci L, Bondì M, Randazzo G, Reggiani C, Natali AN, Pavan PG, Fibre and extracellular matrix contributions to passive forces in human skeletal muscles: an experimental based constitutive law for numerical modelling of the passive element in the classical Hill-type three element model, PLoS One 14 (2019), doi:10.1371/JOURNAL.PONE.0224232. PMC6830811 31689322

[9] Meyer G, Lieber RL, Muscle fibers bear a larger fraction of passive muscle tension in frogs compared with mice, J. Exp. Biol. 221 (2018), doi:10.1242/JEB.182089. PMC6262763 30237238

[10] Wohlgemuth RP, Feitzinger RM, Henricson KE, Dinh DT, Brashear SE, Smith LR, The extracellular matrix of dystrophic mouse diaphragm accounts for the majority of its passive stiffness and is resistant to collagenase digestion, Matrix Biol. Plus 18 (2023) 100131, doi:10.1016/J.MBPLUS.2023.100131. 36970609 PMC10036937

[11] Gillies AR, Smith LR, Lieber RL, Varghese S, Method for decellularizing skeletal muscle without detergents or proteolytic enzymes, Tissue Eng. Part C Methods 17 (2011) 383–389, doi:10.1089/TEN.TEC.2010.0438. 20973753 PMC3065727

[12] Wang K, Mccarter R, Wright J, Beverly J, Ramirez-Mitchell R, Regulation of skeletal muscle stiffness and elasticity by titin isoforms: a test of the segmental extension model of resting tension, Proc. Natl. Acad. Sci. U.S.A. 88 (1991) 7101–7105, doi:10.1073/PNAS.88.16.7101. 1714586 PMC52241

[13] Kjaer M, Role of extracellular matrix in adaptation of tendon and skeletal muscle to mechanical loading, Physiol. Rev. 84 (2004) 649–698, doi:10.1152/physrev.00031.2003. 15044685

[14] Smith LR, Lee KS, Ward SR, Chambers HG, Lieber RL, Hamstring contractures in children with spastic cerebral palsy result from a stiffer extracellular matrix and increased in vivo sarcomere length, J. Physiol. 589 (2011) 2625–2639, doi:10.1113/jphysiol.2010.203364. 21486759 PMC3115830

[15] Nuckolls GH, Kinnett K, Dayanidhi S, Domenighetti AA, Duong T, Hathout Y, Lawlor MW, Lee SSM, Magnusson SP, McDonald CM, McNally EM, Miller NF, Olwin BB, Raghavan P, Roberts TJ, Rutkove SB, Sarwark JF, Senesac CR, Vogel LF, Walter GA, Willcocks RJ, Rymer WZ, Lieber RL, Conference report on contractures in musculoskeletal and neurological conditions, Muscle Nerve 61 (2020) 740–744, doi:10.1002/mus.26845. 32108365 PMC7229996

[16] Skalsky AJ, McDonald CM, Prevention and management of limb contractures in neuromuscular diseases, Phys. Med. Rehabil. Clin. N. Am. 23 (2012) 675–687, doi:10.1016/j.pmr.2012.06.009. 22938881 PMC3482407

[17] Depalle B, Qin Z, Shefelbine SJ, Buehler MJ, Influence of cross-link structure, density and mechanical properties in the mesoscale deformation mechanisms of collagen fibrils, J. Mech. Behav. Biomed. Mater. 52 (2015) 1–13, doi:10.1016/j.jmbbm.2014.07.008. 25153614 PMC4653952

[18] Fratzl P, Misof K, Zizak I, Rapp G, Amenitsch H, Bernstorff S, Fibrillar structure and mechanical properties of collagen, J. Struct. Biol. 122 (1998) 119–122, doi:10.1006/JSBI.1998.3966. 9724612

[19] Fratzl P, Collagen: Structure and Mechanics, Springer, New York, NY, 2008, doi:10.1007/9780387739069.

[20] Svensson RB, Mulder H, Kovanen V, Magnusson SP, Fracture mechanics of collagen fibrils: influence of natural cross-links, Biophys. J. 104 (2013) 2476–2484, doi:10.1016/J.BPJ.2013.04.033. 23746520 PMC3672864

[21] Svensson RB, Hansen P, Hassenkam T, Haraldsson BT, Aagaard P, Kovanen V, Krogsgaard M, Kjaer M, Magnusson SP, Mechanical properties of human patellar tendon at the hierarchical levels of tendon and fibril, J. Appl. Physiol. 112 (2012) 419–426, doi:10.1152/japplphysiol.01172.2011. 22114175

[22] Yang L, van der Werf KO, Dijkstra PJ, Feijen J, Bennink ML, Micromechanical analysis of native and cross-linked collagen type I fibrils supports the existence of microfibrils, J. Mech. Behav. Biomed. Mater. 6 (2012) 148–158, doi:10.1016/j.jmbbm.2011.11.008. 22301184

[23] Buehler MJ, Wong SY, Entropic elasticity controls nanomechanics of single tropocollagen molecules, Biophys. J. 93 (2007) 37–43, doi:10.1529/biophysj.106.102616. 17434941 PMC1914436

[24] Purslow PP, Strain-induced reorientation of an intramuscular connective tissue network: implications for passive muscle elasticity, J. Biomech. 22 (1989) 21–31, doi:10.1016/0021-9290(89)90181–4. 2914969

[25] Purslow PP, The structure and functional significance of variations in the connective tissue within muscle, Compar. Biochem. Physiol. Part A: Mol. Integr. Physiol. 133 (2002) 947–966, doi:10.1016/S1095-6433(02)001411. 12485685

[26] Purslow PP, Trotter JA, The morphology and mechanical properties endomysium in series-fibred muscles: variations with muscle length, J. Muscle Res. Cell. Motil. 15 (1994) 299–308. 7929795 10.1007/BF00123482

[27] Scarr G, Fascial hierarchies and the relevance of crossed-helical arrangements of collagen to changes in the shape of muscles, J. Bodyw. Mov. Ther. 20 (2016) 377–387, doi:10.1016/J.JBMT.2015.09.004. 27210857

[28] Gillies AR, Chapman MA, Bushong EA, Deerinck TJ, Ellisman MH, Lieber RL, High resolution three-dimensional reconstruction of fibrotic skeletal muscle extracellular matrix, J. Physiol. 595 (2017) 1159–1171, doi:10.1113/JP273376. 27859324 PMC5309386

[29] Rowe RWD, Collagen fibre arrangement in intramuscular connective tissue. Changes associated with muscle shortening and their possible relevance to raw meat toughness measurements, Int. J. Food Sci. Technol. 9 (1974) 501–508, doi:10.1111/J.1365-2621.1974.TB01799.X.

[30] Passerieux E, Rossignol R, Letellier T, Delage JP, Physical continuity of the perimysium from myofibers to tendons: involvement in lateral force transmission in skeletal muscle, J. Struct. Biol. 159 (2007) 19–28, doi:10.1016/J.JSB.2007.01.022. 17433715

[31] Screen HRC, Bader DL, Lee DA, Shelton JC, Local strain measurement within tendon, Strain 40 (2004) 157–163.

[32] Buckley MR, Sarver JJ, Freedman BR, Soslowsky LJ, The dynamics of collagen uncrimping and lateral contraction in tendon and the effect of ionic concentration, J. Biomech. 46 (2013) 2242–2249, doi:10.1016/j.jbiomech.2013.06.029. 23876711 PMC3769524

[33] Pins GD, Christiansen DL, Patel R, Silver FH, Self-assembly of collagen fibers. Influence of fibrillar alignment and decorin on mechanical properties, Biophys. J. 73 (1997) 2164–2172, doi:10.1016/S0006-3495(97)78247X. 9336212 PMC1181117

[34] Boorman RS, Norman T, Matsen FA, Clark JM, Using a freeze substitution fixation technique and histological crimp analysis for characterizing regions of strain in ligaments loaded in situ, J. Orthop. Res. 24 (2006) 793–799, doi:10.1002/jor.20081. 16514649

[35] Brashear SE, Wohlgemuth RP, Gonzalez G, Smith LR, Passive stiffness of fibrotic skeletal muscle in mdx mice relates to collagen architecture, J. Physiol. 599 (2021) 943–962, doi:10.1113/JP280656. 33247944 PMC9926974

[36] Brashear SE, Wohlgemuth RP, Hu L-YR, Jbeily EH, Christiansen BA, Smith LR, Collagen cross-links scale with passive stiffness in dystrophic mouse muscles, but are not altered with administration of lysyl oxidase inhibitor, PLoS ONE 17 (2022) e0271776, doi:10.1101/2022.07.08.499292. 36302059 PMC9612445

[37] Sahani R, Wallace CH, Jones BK, Blemker SS, Diaphragm muscle fibrosis involves changes in collagen organization with mechanical implications in Duchenne Muscular Dystrophy, J. Appl. Physiol. (Bethesda, Md. : 1985) (2022), doi:10.1152/JAPPLPHYSIOL.00248.2021. PMC9076426 35050792

[38] Hammers DW, Hart CC, Matheny MK, Wright LA, Armellini M, Barton ER, Sweeney HL, The D2.mdx mouse as a preclinical model of the skeletal muscle pathology associated with Duchenne muscular dystrophy, Sci. Rep. 10 (2020) 1–12, doi:10.1038/s41598020-70987y. 32826942 PMC7442653

[39] McGreevy JW, Hakim CH, McIntosh MA, Duan D, Animal models of Duchenne muscular dystrophy: from basic mechanisms to gene therapy, Dis. Model. Mech. 8 (2015) 195–213, doi:10.1242/dmm.018424. 25740330 PMC4348559

[40] Van Putten M, Putker K, Overzier M, Adamzek WA, Pasteuning-Vuhman S, Plomp JJ, Aartsma-Rus A, Natural disease history of the D2-mdx mouse model for Duchenne muscular dystrophy, FASEB 33 (2019) 8110–8124, doi:10.1096/fj.201802488R. PMC6593893 30933664

[41] Gordish-Dressman H, Willmann R, Dalle Pazze L, Kreibich A, van Putten M, Heydemann A, Bogdanik L, Lutz C, Davies K, Demonbreun AR, Duan D, Elsey D, Fukada S, Girgenrath M, Patrick Gonzalez J, Grounds MD, Nichols A, Partridge T, Passini M, Sanarica F, Schnell FJ, Wells DJ, Yokota T, Young CS, Zhong Z, Spurney C, Spencer M, De Luca A, Nagaraju K, Aartsma-Rus A, “Of mice and measures”: a project to improve how we advance duchenne muscular dystrophy therapies to the clinic, J. Neuromuscul. Dis. 5 (n.d.) 407–417. 10.3233/JND-180324. PMC6218134 30198876

[42] Hart CC, il Lee Y, Xie J, Gao G, Lin BL, Hammers DW, Sweeney HL, Potential limitations of microdystrophin gene therapy for Duchenne muscular dystrophy, JCI Insight 9 (2024), doi:10.1172/jci.insight.165869. PMC11382885 38713520

[43] Mendez J, Keys A, Density and composition of mammalian muscle, Metabolism (1960) 184–188.

[44] Ward SR, Tomiya A, Regev GJ, Thacker BE, Benzl RC, Kim CW, Lieber RL, Passive mechanical properties of the lumbar multifidus muscle support its role as a stabilizer, J. Biomech. 42 (2009) 1384–1389, doi:10.1016/J.JBIOMECH.2008.09.042. 19457491 PMC2752430

[45] Hakim CH, Grange RW, Duan D, The passive mechanical properties of the extensor digitorum longus muscle are compromised in 2-to 20-mo-old mdx mice, J. Appl. Physiol. 110 (2011) 1656–1663, doi:10.1152/japplphysiol.01425.2010. 21415170 PMC3119138

[46] Smith LR, Barton ER, Collagen content does not alter the passive mechanical properties of fibrotic skeletal muscle in mdx mice, Am. J. Physiol., Cell Physiol. 306 (2014), doi:10.1152/AJPCELL.00383.2013. PMC4024713 24598364

[47] Roche SM, Gumucio JP, Brooks SV, Mendias CL, Claflin DR, Measurement of maximum isometric force generated by permeabilized skeletal muscle fibers, JoVE (J. Vis. Exp.) 2015 (2015) e52695. 10.3791/52695. PMC4545153 26131687

[48] Hu LY, Mileti CJ, Loomis T, Brashear SE, Ahmad S, Chellakudam RR, Wohlgemuth RP, Gionet-Gonzales MA, Leach JK, Smith LR, Skeletal muscle progenitors are sensitive to collagen architectural features of fibril size and cross linking, Am. J. Physiol. - Cell Physiol. 321 (2021) C330–C342, doi:10.1152/ajpcell.00065.2021. 34191625 PMC8424678

[49] Moreaux L, Sandre O, Charpak S, Blanchard-Desce M, Mertz J, Coherent scattering in multi-harmonic light microscopy, Biophys. J. 80 (2001) 1568–1574, doi:10.1016/S0006-3495(01)761292. 11222317 PMC1301348

[50] Turcotte R, Mattson JM, Wu JW, Zhang Y, Lin CP, Molecular order of arterial collagen using circular polarization second-harmonic generation imaging, Biophys. J. 110 (2016) 530–533, doi:10.1016/j.bpj.2015.12.030. 26806883 PMC4744203

[51] Flesch M, Schiffer F, Zolk O, Pinto Y, Rosenkranz S, Hirth-Dietrich C, Arnold G, Paul M, Bohm M, Contractile systolic and diastolic dysfunction in renin-induced hypertensive cardiomyopathy, Hypertension 30 (1997) 383–391, doi:10.1161/01.HYP.30.3.383/FORMAT/EPUB. 9314421

[52] Saini K, Cho S, Dooling LJ, Discher DE, Tension in fibrils suppresses their enzymatic degradation – A molecular mechanism for ‘use it or lose it, Matrix Biology 85–86 (2020) 34–46, doi:10.1016/j.matbio.2019.06.001. PMC6906264 31201857

[53] Gaul RT, Nolan DR, Ristori T, Bouten CVC, Loerakker S, Lally C, Strain mediated enzymatic degradation of arterial tissue: insights into the role of the non-collagenous tissue matrix and collagen crimp, Acta Biomater. 77 (2018) 301–310, doi:10.1016/J.ACTBIO.2018.06.037. 30126592

[54] Flynn BP, Bhole AP, Saeidi N, Liles M, Dimarzio CA, Ruberti JW, Mechanical strain stabilizes reconstituted collagen fibrils against enzymatic degradation by mammalian collagenase matrix metalloproteinase 8 (MMP-8), PLoS One 5 (2010) e12337, doi:10.1371/journal.pone.0012337. 20808784 PMC2925882

[55] Smith LR, Hammers DW, Lee Sweeney H, Barton ER, Increased collagen cross-linking is a signature of dystrophin-deficient muscle, Muscle Nerve 54 (2016) 71–78, doi:10.1002/mus.24998. 26616495 PMC5067682

[56] Schüler SC, Kirkpatrick JM, Schmidt M, Santinha D, Koch P, Di Sanzo S, Cirri E, Hemberg M, Ori A, von Maltzahn J, Extensive remodeling of the extracellular matrix during aging contributes to age-dependent impairments of muscle stem cell functionality, Cell Rep. 35 (2021) 109223, doi:10.1016/j.celrep.2021.109223. 34107247

[57] Lofaro FD, Cisterna B, Lacavalla MA, Boschi F, Malatesta M, Quaglino D, Zancanaro C, Boraldi F, Age-related changes in the matrisome of the mouse skeletal muscle, Int. J. Mol. Sci. 22 (2021) 10564, doi:10.3390/IJMS221910564. 34638903 PMC8508832

[58] Wohlgemuth RP, Brashear SE, Smith LR, Alignment, cross linking, and beyond: a collagen architect’s guide to the skeletal muscle extracellular matrix, Am. J. Physiol. Cell Physiol. 325 (2023) C1017–C1030, doi:10.1152/ajpcell.00287.2023. 37661921 PMC10635663

[59] Yanagishita M, Function of proteoglycans in the extracellular matrix, Acta Pathol. Jpn. 43 (1993) 283–293, doi:10.1111/J.1440-1827.1993.TB02569.X. 8346704

[60] Ameye L, Young MF, Mice deficient in small leucine-rich proteoglycans: novel in vivo models for osteoporosis, osteoarthritis, Ehlers-Danlos syndrome, muscular dystrophy, and corneal diseases, Glycobiology 12 (2002) 107–116. 10.1093/glycob/cwf065 12213783

[61] Weber IT, Harrison RW, Iozzo RV, Model structure of decorin and implications for collagen fibrillogenesis, J. Biol. Chem. 271 (1996) 31767–31770, doi:10.1074/jbc.271.50.31767. 8943211

[62] Häkkinen L, Strassburger S, Kähäri VM, Scott PG, Eichstetter I, Iozzo RV, Larjava H, A role for decorin in the structural organization of periodontal ligament, Lab. Investig. 80 (2000) 1869–1880 200012 80, doi:10.1038/labinvest.3780197. 11140699

[63] Halper J, Kjaer M, Basic components of connective tissues and extracellular matrix: elastin, fibrillin, fibulins, fibrinogen, fibronectin, laminin, tenascins and thrombospondins, Adv. Exp. Med. Biol. 802 (2014) 31–47, doi:10.1007/978-94-00778931_3. 24443019

[64] Sanes JR, The basement membrane/basal lamina of skeletal muscle, J. Biol. Chem. 278 (2003) 12601–12604, doi:10.1074/jbc.R200027200. 12556454

[65] Grounds MD, Sorokin L, White J, Strength at the extracellular matrix–muscle interface, Scand. J. Med. Sci. Sports 15 (2005) 381–391, doi:10.1111/J.1600-0838.2005.00467.X. 16293150

[66] Gosselin LE, Martinez DA, Vailas AC, Sieck GC, Passive length-force properties of senescent diaphragm: relationship with collagen characteristics, J. Appl. Physiol. (Bethesda, Md. : 1985) 76 (1994) 2680–2685, doi:10.1152/JAPPL.1994.76.6.2680. 7928900

[67] Palokangas H, Kovanen V, Duncan R, Robins SP, Age-related changes in the concentration of hydroxypyridinium crosslinks in functionally different skeletal muscles, Matrix 12 (1992) 291–296, doi:10.1016/S0934-8832(11)800818. 1435513

[68] Olson LC, Redden JT, Schwartz Z, Cohen DJ, McClure MJ, Advanced glycation end-products in skeletal muscle aging, Bioengineering 2021, Vol. 8, Page 168 8 (2021) 168. 10.3390/BIOENGINEERING8110168. 34821734 PMC8614898

[69] Olson LC, Nguyen TM, Heise RL, Boyan BD, Schwartz Z, McClure MJ, Advanced glycation end products are retained in decellularized muscle matrix derived from aged skeletal muscle, Int. J. Mol. Sci. 22 (2021), doi:10.3390/IJMS22168832. PMC8396213 34445538

[70] Haus JM, Carrithers JA, Trappe SW, Trappe TA, Collagen, cross-linking, and advanced glycation end products in aging human skeletal muscle, J. Appl. Physiol. 103 (2007) 2068–2076, doi:10.1152/japplphysiol.00670.2007.-We. 17901242

[71] Trombitás K, Wu Y, McNabb M, Greaser M, Kellermayer MSZ, Labeit S, Granzier H, Molecular basis of passive stress relaxation in human soleus fibers: assessment of the role of immunoglobulin-like domain unfolding, Biophys. J. 85 (2003) 3142–3153, doi:10.1016/S0006-3495(03)747328. 14581214 PMC1303590

[72] Calvo B, Sierra M, Grasa J, Muñoz MJ, Peña E, Determination of passive viscoelastic response of the abdominal muscle and related constitutive modeling: stress-relaxation behavior, J. Mech. Behav. Biomed. Mater. 36 (2014) 47–58, doi:10.1016/j.jmbbm.2014.04.006. 24793173

[73] Heydemann A, Huber JM, Demonbreun A, Hadhazy M, McNally EM, Genetic background influences muscular dystrophy, Neuromusc. Disord. 15 (2005) 601–609, doi:10.1016/J.NMD.2005.05.004. 16084087

[74] Heydemann A, Ceco E, Lim JE, Hadhazy M, Ryder P, Moran JL, Beier DR, Palmer AA, McNally EM, Latent TGF-beta-binding protein 4 modifies muscular dystrophy in mice, J. Clin. Investig. 119 (2009) 3703–3712, doi:10.1172/JCI39845. 19884661 PMC2786802

[75] Smith LR, Barton ER, Regulation of fibrosis in muscular dystrophy, Matrix Biol. : J. Int. Soc. Matrix Biol. 68–69 (2018) 602, doi:10.1016/J.MATBIO.2018.01.014. PMC6519730 29408413

[76] Minayoshi Y, Maeda H, Hamasaki K, Nagasaki T, Takano M, Fukuda R, Mizuta Y, Tanaka M, Sasaki Y, Otagiri M, Watanabe H, Maruyama T, Mouse type-I interferon-mannosylated albumin fusion protein for the treatment of chronic hepatitis, Pharmaceuticals (Basel) 17 (2024) 260, doi:10.3390/ph17020260. 38399475 PMC10893114

[77] Purohit A, Kandiyal B, Kumar S, Pragasam AK, Kamboj P, Talukdar D, Verma J, Sharma V, Sarkar S, Mahajan D, Yadav R, Ahmed R, Nanda R, Dikshit M, Banerjee SK, null Shalimar B. Das, Collinsella aerofaciens linked with increased ethanol production and liver inflammation contribute to the pathophysiology of NAFLD, iScience 27 (2024) 108764, doi:10.1016/j.isci.2023.108764. 38313048 PMC10837629

[78] Selvarani R, Van Michelle Nguyen H, Thadathil N, Wolf RF, Freeman WM, Wiley CD, Deepa SS, Richardson A, Characterization of novel mouse models to study the role of necroptosis in aging and age-related diseases, Geroscience 45 (2023) 3241–3256, doi:10.1007/s11357023009557. 37792157 PMC10643444

[79] Liang L, Jones C, Chen S, Sun B, Jiao Y, Heterogeneous force network in 3D cellularized collagen networks, Phys. Biol. 13 (2016) 066001, doi:10.1088/1478-3975/13/6/066001. 27779119

[80] Ban E, Franklin JM, Nam S, Smith LR, Wang H, Wells RG, Chaudhuri O, Liphardt JT, Shenoy VB, Mechanisms of plastic deformation in collagen networks induced by cellular forces, Biophys. J. 114 (2018) 450–461, doi:10.1016/J.BPJ.2017.11.3739. 29401442 PMC5984980

[81] Zhang S, Cao X, Stablow AM, Shenoy VB, Winkelstein BA, Tissue strain reorganizes collagen with a switchlike response that regulates neuronal extracellular signal-regulated kinase phosphorylation in vitro: implications for ligamentous injury and mechanotransduction, J. Biomech. Eng. 138 (2016) 021013, doi:10.1115/1.4031975. 26549105 PMC4844078

[82] Purslow PP, The structure and role of intramuscular connective tissue in muscle function, Front. Physiol. 11 (2020) 495, doi:10.3389/FPHYS.2020.00495. 32508678 PMC7248366

[83] Reyna WE, Pichika R, Ludvig D, Perreault EJ, Efficiency of skeletal muscle decellularization methods and their effects on the extracellular matrix, J. Biomech. 110 (2020), doi:10.1016/J.JBIOMECH.2020.109961. PMC7487037 32827769

